# N-terminal and mid-region tau fragments as fluid biomarkers in neurological diseases

**DOI:** 10.1093/brain/awab481

**Published:** 2022-07-27

**Authors:** Anniina Snellman, Juan Lantero-Rodriguez, Andreja Emeršič, Agathe Vrillon, Thomas K Karikari, Nicholas J Ashton, Milica Gregorič Kramberger, Saša Čučnik, Claire Paquet, Uroš Rot, Henrik Zetterberg, Kaj Blennow

**Affiliations:** Department of Psychiatry and Neurochemistry, Institute of Neuroscience and Physiology, The Sahlgrenska Academy at the University of Gothenburg, Mölndal, Sweden; Turku PET Centre, University of Turku, Turku, Finland; Department of Psychiatry and Neurochemistry, Institute of Neuroscience and Physiology, The Sahlgrenska Academy at the University of Gothenburg, Mölndal, Sweden; Department of Neurology, University Medical Centre Ljubljana, Ljubljana, Slovenia; Faculty of Pharmacy, University of Ljubljana, Ljubljana, Slovenia; Université de Paris, Cognitive Neurology Center, GHU Nord APHP Hospital Lariboisière Fernand Widal, Paris, France; Université de Paris, Inserm UMR S11-44 Therapeutic Optimization in Neuropsychopharmacology, Paris, France; Department of Psychiatry and Neurochemistry, Institute of Neuroscience and Physiology, The Sahlgrenska Academy at the University of Gothenburg, Mölndal, Sweden; Department of Psychiatry, University of Pittsburgh, Pittsburgh, PA, USA; Department of Psychiatry and Neurochemistry, Institute of Neuroscience and Physiology, The Sahlgrenska Academy at the University of Gothenburg, Mölndal, Sweden; Wallenberg Centre for Molecular and Translational Medicine, University of Gothenburg, Gothenburg, Sweden; Department of Old Age Psychiatry, Maurice Wohl Clinical Neuroscience Institute, King’s College London, London, UK; NIHR Biomedical Research Centre for Mental Health and Biomedical Research Unit for Dementia at South London and Maudsley NHS Foundation, London, UK; Department of Neurology, University Medical Centre Ljubljana, Ljubljana, Slovenia; Faculty of Medicine, University of Ljubljana, Ljubljana, Slovenia; Department of Neurology, University Medical Centre Ljubljana, Ljubljana, Slovenia; Faculty of Pharmacy, University of Ljubljana, Ljubljana, Slovenia; Department of Rheumatology, University Medical Centre Ljubljana, Ljubljana, Slovenia; Université de Paris, Cognitive Neurology Center, GHU Nord APHP Hospital Lariboisière Fernand Widal, Paris, France; Université de Paris, Inserm UMR S11-44 Therapeutic Optimization in Neuropsychopharmacology, Paris, France; Department of Neurology, University Medical Centre Ljubljana, Ljubljana, Slovenia; Faculty of Medicine, University of Ljubljana, Ljubljana, Slovenia; Department of Psychiatry and Neurochemistry, Institute of Neuroscience and Physiology, The Sahlgrenska Academy at the University of Gothenburg, Mölndal, Sweden; Clinical Neurochemistry Laboratory, Sahlgrenska University Hospital, Mölndal, Sweden; Department of Neurodegenerative Disease, UCL Institute of Neurology, Queen Square, London, UK; UK Dementia Research Institute at UCL, London, UK; Hong Kong Center for Neurodegenerative Diseases, Hong Kong, China; Department of Psychiatry and Neurochemistry, Institute of Neuroscience and Physiology, The Sahlgrenska Academy at the University of Gothenburg, Mölndal, Sweden; Clinical Neurochemistry Laboratory, Sahlgrenska University Hospital, Mölndal, Sweden

**Keywords:** Alzheimer’s disease, tau, biomarker, cerebrospinal fluid, plasma

## Abstract

Brain-derived tau secreted into CSF and blood consists of different N-terminal and mid-domain fragments, which may have a differential temporal course and thus, biomarker potential across the Alzheimer’s disease continuum or in other neurological diseases. While current clinically validated total tau assays target mid-domain epitopes, comparison of these assays with new biomarkers targeting N-terminal epitopes using the same analytical platform may be important to increase the understanding of tau pathophysiology.

We developed three total tau immunoassays targeting specific N-terminal (NTA and NTB total tau) or mid-region (MR total tau) epitopes, using single molecule array technology. After analytical validation, the diagnostic performance of these biomarkers was evaluated in CSF and compared with the Innotest total tau (and as proof of concept, with N-p-tau181 and N-p-tau217) in three clinical cohorts (*n* = 342 total). The cohorts included participants across the Alzheimer’s disease continuum (*n* = 276), other dementias (*n* = 22), Creutzfeldt–Jakob disease (*n* = 24), acute neurological disorders (*n* = 18) and progressive supranuclear palsy (*n* = 22). Furthermore, we evaluated all three new total tau biomarkers in plasma (*n* = 44) and replicated promising findings with NTA total tau in another clinical cohort (*n* = 50).

In CSF, all total tau biomarkers were increased in Alzheimer’s disease compared with controls (*P* < 0.0001) and correlated with each other (*r_s_* = 0.53−0.95). NTA and NTB total tau, but not other total tau assays, distinguished amyloid-positive and amyloid-negative mild cognitive impairment with high accuracies (AUCs 84% and 82%, *P* < 0.001) matching N-p-tau217 (AUC 83%; DeLong test *P* = 0.93 and 0.88). All total tau assays were excellent in differentiating Alzheimer’s disease from other dementias (*P* < 0.001, AUCs 89–100%). In Creutzfeldt–Jakob disease and acute neurological disorders, N-terminal total tau biomarkers had significantly higher fold changes versus controls in CSF (45–133-fold increase) than Innotest or MR total tau (11–42-fold increase, *P* < 0.0001 for all). In progressive supranuclear palsy, CSF concentrations of all total tau biomarkers were similar to those in controls. Plasma NTA total tau concentrations were increased in Alzheimer’s disease compared with controls in two independent cohorts (*P* = 0.0056 and 0.0033), while Quanterix total tau performed poorly (*P* = 0.55 and 0.44).

Taken together, N-terminal-directed CSF total tau biomarkers increase ahead of standard total tau alternatives in the Alzheimer’s disease continuum, increase to higher degrees in Creutzfeldt–Jakob disease and acute neurological diseases and show better potential than Quanterix total tau as Alzheimer’s disease blood biomarkers. For progressive supranuclear palsy, other tau biomarkers should continue to be investigated.

## Teaser

Snellman and Lantero-Rodriguez *et al*. develop new N-terminal-directed CSF total tau assays. They show that levels of these biomarkers both increase ahead of standard t-tau in patients on the Alzheimer’s disease continuum, and are higher in patients with CJD or acute neurological diseases compared to standard t-tau.

## Introduction

Tauopathy is an umbrella term used to classify neurodegenerative diseases in which misfolded and aggregated tau protein constitutes the key pathology.^1^ The molecular mechanisms behind tauopathies such as Alzheimer’s disease, progressive supranuclear palsy and frontotemporal dementia are likely distinct and, additionally, they differ in terms of clinical presentation, anatomical distribution and cell types affected by the tau aggregates.^1^ Although a definitive diagnosis of a tauopathy requires neuropathological examination post-mortem to confirm the presence and distribution of specific tau accumulations in the brain, differential diagnosis can be aided by CSF biomarkers.^2,3^

CSF total tau (t-tau), referring to methods reacting to both phosphorylated and non-phosphorylated tau isoforms, is an established core Alzheimer’s disease biomarker^2^ that, together with amyloid-β (Aβ_1-42_ or Aβ_1-42_/Aβ_1-40_) and phosphorylated tau at threonine 181 (p-tau181), is used for biological definition of Alzheimer’s disease, in accordance with the amyloid/tau/neurodegeneration (A/T/N) classification framework.^4^ The first CSF t-tau assay was developed in the 1990s, and traditionally, increased CSF t-tau has been suggested to reflect tau release due to neuronal injury and/or axonal degeneration.^5^ This interpretation was supported by the rapid increases of CSF t-tau seen also upon acute neuronal injury, such as stroke^6^ or brain trauma,^7,8^ and in diseases with aggressive neurodegeneration, such as Creutzfeldt–Jakob disease (CJD).^9^ However, since normal CSF t-tau levels are typically detected in non-Alzheimer’s disease dementias and primary tauopathies commonly absent of Aβ pathology,^3,10^ the increase in Alzheimer’s disease has been hypothesized to be caused at least partly by a change in tau metabolism in neurons affected by Aβ toxicity,^11^ possibly from the dystrophic neurites surrounding the plaques.

Immunoassays measuring t-tau in clinical routine, such as the fully automated Elecsys and Lumipulse methods, utilize antibodies targeting epitopes located in the mid-region of the protein.^12,13^ Since these assays can recognize all six tau isoforms, they are stated to measure ‘total tau’. However, in addition to its various post-translational modifications,^14,15^ soluble tau is known to exist in proteolytic fragments of different lengths,^16^ and shorter N-terminal fragments lacking the mid-region cannot be detected by the classic t-tau assays. Previous work targeting different N-terminal epitopes of tau in CSF have shown that these fragments are increased in Alzheimer’s disease at the mild cognitive impairment (MCI) and dementia stages.^17–19^ In addition, p-tau assays targeting N-terminal fragments phosphorylated at threonine-181 (N-p-tau181) and threonine-217 (N-p-tau217) have shown earlier abnormal levels in CSF in comparison to mid-region p-tau181 across the Alzheimer’s disease continuum.^20,21^

**Figure 1:**
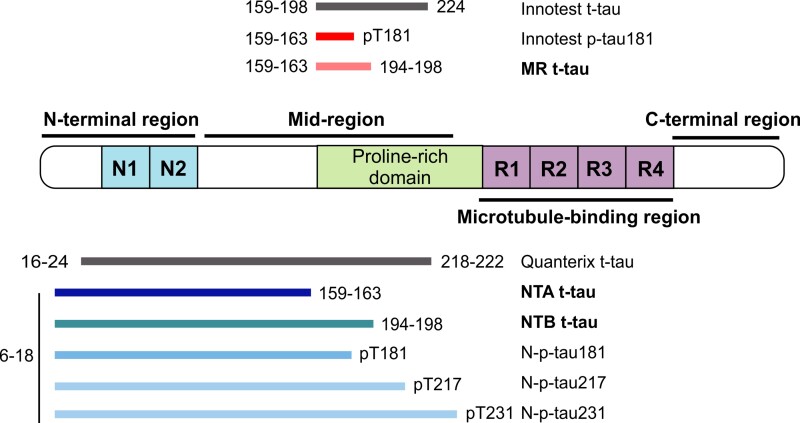
**Schematic presentation of all t-tau and p-tau biomarkers included in the study.** Immunoassays developed during this study are presented in bold font.

Recently, tau was shown to consist of N-terminal and mid-region species in CSF and predominantly N-terminal forms in blood.^19,22,23^ This knowledge has been applied to develop new p-tau immunoassays for use in blood, targeting different epitopes than the validated assays. In blood, N-p-tau181, N-p-tau217 and N-p-tau231 biomarkers (Fig. 1) are increased early in the Alzheimer’s disease continuum starting from the preclinical stage, and correlate well with CSF p-tau, t-tau, amyloid PET and tau PET.^21,24–27^ Similarly, a commercial Simoa t-tau assay (the most widely used blood t-tau biomarker) targeting N-terminal-to-mid-region epitopes is widely available. While CSF t-tau (directed at mid-region epitopes; Fig. 1) is an established Alzheimer’s disease biomarker, the plasma t-tau alternative is only marginally increased in Alzheimer’s disease compared with controls, shows large overlap between diagnostic groups, and correlates poorly with CSF t-tau.^28–30^ It also does not change in relation to grey matter volume loss, cross-sectionally or longitudinally.^30^ Recently, an N-terminal-targeted plasma t-tau alternative (NT1 tau) was seen to be increased in Alzheimer’s disease at the MCI and dementia stages versus controls,^18^ and in individuals who later progressed to dementia.^31^ These findings, in agreement with p-tau data, suggest that truncation leading to shorter N-terminal fragments of tau is an early event in Alzheimer’s disease pathophysiology and that N-terminal tau forms might provide superior biomarker performance in plasma. However, it is unclear how different tau fragments compare as neurodegeneration markers in the Alzheimer’s disease continuum and other neurodegenerative disorders.

The aim of this study was to develop and validate novel t-tau immunoassays targeting N-terminal and mid-region epitopes using Simoa technology and subsequently investigate the levels of these new biomarkers versus clinically validated mid-region t-tau, p-tau and N-p-tau assays in CSF in clinical cohorts across the Alzheimer’s disease continuum, as well as across other neurological diseases including those known for high (e.g. CJD and a heterogeneous group of other acute neurological disorders) and normal (e.g. progressive supranuclear palsy) t-tau levels. In addition, we performed an exploratory analysis in plasma, evaluating the biomarker potential of the different t-tau immunoassays versus the Quanterix t-tau in two independent cohorts.

## Materials and methods

### Study design

This was a cross-sectional, observational study conducted in collaboration with three centres (The Sahlgrenska Academy at the University of Gothenburg, Gothenburg, Sweden; University Medical Centre Ljubljana, Ljubljana, Slovenia; and Université de Paris, Paris, France).

### Immunoassay development and validation

Based on our recent success in developing N-terminal-directed N-p-tau181, N-p-tau217 and N-p-tau231 biomarkers,^20,21,24,26,32^ we developed two immunoassays targeting N-terminal-bearing tau fragments: (i) NTA t-tau, containing the N-terminal epitope mapped between amino acids (aa) 6–18 and 159–163; and (ii) NTB t-tau, containing epitopes at aa 6–18 and 194–198. In addition, we developed an assay targeting the mid-region aa 159–163 and 194–198 epitopes (MR t-tau) as a Simoa-based replica of the Innotest t-tau assay to enable direct comparison on the same analytical platform. For the NTA and MR t-tau immunoassays, mouse monoclonal antibody targeting aa 159–163 (HT7, #MN1000, Thermo Scientific) was used as a capture antibody. Mouse monoclonal antibodies targeting aa 6–18 (Tau12, #806502, BioLegend) and aa 194–198 (BT2, #MN1010, Thermo Scientific) were used as detectors, respectively. For the NTB t-tau assay, mouse monoclonal antibodies targeting aa 6–18 (1–100, #816601, BioLegend) and aa 194–198 (BT2, #MN1010, Thermo Scientific) were used for capture and detection, respectively. Recombinant non-phosphorylated full-length Tau-441 (#T08-54N, SignalChem) was used as a calibrator in all the in-house t-tau assays. After optimizing the assay protocols, quantification limits, dilution linearity, precision, accuracy, and spike recovery were assessed for all three assays. More detailed description of the immunoassay development and validation processes are available in the Supplementary material.

### Studied biomarkers

All biomarkers studied in this study are presented in Fig. 1. Analytical and clinical details of the Innotest® hTau Ag enzyme-linked immunosorbent assay (ELISA; t-tau; Fujirebio), Innotest® Phosphotau (181P) ELISA (Fujirebio), in-house N-p-tau181 and in-house N-p-tau217 Simoa measurements from the clinical CSF cohort have been reported previously^20^ and were included to enable direct comparison between the classical mid-region (t-tau and p-tau181), N-t-tau and N-p-tau biomarkers. Plasma samples were analysed with commercial t-tau assays from Quanterix, i.e. the single-analyte Tau 2.0 kit (#101552) for the pilot cohort and the multiplexed Neurology 3-Plex kit (#101995), in-house N-p-tau181 and N-p-tau231 for the clinical cohort using previously published methods.^24,26^ Note that both of the Quanterix t-tau assays target identical epitopes.

### Participants for CSF total tau study

#### Pilot cohort

The pilot study included samples from CSF biomarker-positive Alzheimer’s disease patients (*n* = 22) and biomarker-negative control patients (*n* = 22) clinically assessed in the Sahlgrenska University Hospital, Gothenburg, Sweden. Patients included in the Alzheimer’s disease group were assessed due to suspected Alzheimer’s disease, and no evidence of other neurological conditions was present. Patients in the neurological control group had consulted the clinic due to minor neurological or psychiatric symptoms, but those with a diagnosis of neurocognitive disorder were excluded, and Alzheimer’s disease core CSF biomarkers were within normal range. The groups were defined by their core Alzheimer’s disease CSF biomarker profiles (CSF Aβ_42_ < 530 ng/l, CSF p-tau181 > 60 ng/l and CSF t-tau > 350 ng/l for Alzheimer’s disease and levels within normal range for control patients).

#### Clinical cohort 1: the Alzheimer’s continuum

To study the profiles of different t-tau biomarkers in Alzheimer’s disease continuum, a clinic-based prospective memory centre cohort from the University Medical Centre, Ljubljana, Slovenia, was used. The cohort included individuals with biologically-defined Alzheimer’s disease, showing both biomarker positivity and clinical presentation [*n* = 115; A+/T+/N+, mini-mental state examination (MMSE) score = 21(16–25)], Aβ positive MCI [*n* = 33, A+/T−/N−, MMSE = 26(24–27)], Aβ negative MCI [*n* = 58; A−/T−/N−, MMSE = 27(26–28)], non-Alzheimer’s disease dementia [*n* = 22; A−/T−/N−, MMSE = 21(19–23)] and Aβ− cognitively unimpaired neurological controls [*n* = 26; A−/T−/N−, MMSE = 29(29–30)]. The control group presented with subjective cognitive concerns or sensory disturbances but had a normal CSF Alzheimer’s disease biomarker profile. The non-Alzheimer’s disease dementia group included individuals with a diagnosis of alcohol-related dementia (*n* = 3), vascular dementia (*n* = 4), mixed vascular and non-Alzheimer’s disease cortical dementia (*n* = 7) and unspecified dementia (*n* = 8). A/T/N profiles were defined by core Alzheimer’s disease CSF biomarkers with local cut-offs (CSF Aβ_42_ < 570 ng/l, CSF Aβ_42/40_ < 0.07, CSF p-tau181 > 60 ng/l and CSF t-tau > 400 ng/l).

#### Clinical cohort 2: other neurodegenerative disorders

In addition to Alzheimer’s disease, we investigated individuals with a diagnosis of either CJD (*n* = 24), acute neurological disorders [*n* = 18, including individuals with status epilepticus (*n* = 9), ischemic stroke (*n* = 7), hepatic encephalopathy (*n* = 1), and limbic encephalitis (*n* = 1)] or progressive supranuclear palsy (*n* = 22) from the University Medical Centre, Ljubljana, Slovenia. Comparison between groups included the Alzheimer’s disease and neurological control groups described above.

### Participants for plasma total tau study

#### Pilot cohort

The plasma pilot cohort included samples from CSF biomarker-positive Alzheimer’s disease patients (*n* = 22) and biomarker negative control patients (*n* = 22) assessed in the Sahlgrenska University Hospital, Gothenburg, Sweden. Groups were defined as described above for the pilot CSF cohort.

#### Clinical cohort

Pilot findings with plasma NTA t-tau were replicated using a clinic-based prospective memory centre cohort, the BioCogBank Paris Lariboisiére Cohort (Paris, France). CSF biomarker results, neuropsychological assessment, brain MRI and respective diagnostic criteria were used to establish reliable diagnosis of Alzheimer’s disease and other disorders. The cohort included patients with Alzheimer’s disease [*n* = 19, A+/T+/N+, MMSE = 19 (13–24)], Aβ-positive MCI [*n* = 6, A+/T+/N+, MMSE = 23 (22–26)], Aβ-negative MCI [*n* = 13, A−/T−/N−, MMSE = 25 (24–27)], non-Alzheimer’s disease dementia [*n* = 3, MMSE = 24 (17–28)] and neurological controls [*n* = 8, A−/T−/N−, MMSE = 28 (26–30)]. The non-Alzheimer’s disease dementia group included individuals with a diagnosis of vascular (*n* = 1, A−/T−/N−) or frontotemporal dementia (*n* = 2, A−/T−/N+ and A−/T+/N+). A/T/N profiles were defined by core Alzheimer’s disease CSF biomarkers with local cut-offs (Lumipulse CSF Aβ_42/40_ < 0.061, CSF p-tau181 > 61 ng/l, CSF t-tau > 469 ng/l).

### Informed consent

Ethical permission was obtained from the ethics committee at the university of Gothenburg (#EPN140811, pilot CSF/plasma cohorts), the Ministry of Health, Republic of Slovenia (0120-442/2017/3, clinical CSF cohorts 1 and 2) and the Bichat Hospital at the Paris University (no. 10-037 18/03/2010, clinical plasma cohort). Informed consent was obtained from all participants according to the Declaration of Helsinki.

### Biomarker measurements

All MR, NTA and NTB t-tau measurements were performed in the Neurochemistry laboratory at University of Gothenburg (Mölndal, Sweden) using Simoa HD-X or HD-1 instruments (Quanterix) between December 2020 and June 2021. Before measurements were taken, assay beads and helper beads were suspended in bead diluent, biotinylated detector antibodies in Tau 2.0 assay buffer (#101556, Quanterix) and the enzyme streptavidin-conjugated β-galactosidase (SBG) concentrate (#103397, Quanterix) in SBG diluent (#100376, Quanterix). For CSF, randomized samples were thawed, vortexed briefly, plated and diluted 1:4 in Tau 2.0 assay diluent. Additional measurements with 1:10 dilution were needed for a subset of CJD (7/24 for NTA, 5/24 for NTB, 11/24 for MR t-tau) and acute neurological disorders samples (3/18 for NTA, 1/18 for NTB, 1/18 for MR t-tau) due to extremely high signals. For plasma, randomized samples were thawed, vortexed, centrifuged (4000*g*, 10 min) and diluted 1:2 with Tau 2.0 assay diluent. An eight-point calibrator curve from recombinant non-phosphorylated full-length Tau-441, and two internal quality control (iQC) samples were included on each plate before and after the analysed samples to control for inter and intra-assay variability. Calibrators and internal quality control samples were run as duplicates and CSF/plasma samples as singlicates in each plate. For commercial t-tau measurements, reagents and samples were prepared following the manufacturer’s instructions. Further methodological details can be found in the Supplementary material.

### Statistical analysis

Data are presented as median (interquartile range). Statistical analyses were performed with GraphPad Prism v. 9.0.1 (GraphPad, San Diego, California, USA) and MedCalc (Ostend, Belgium). The normality of the data was inspected visually and with the D’Agostino and Pearson normality test, and because not all biomarker data followed Gaussian distribution even after transformation, non-parametric tests were used. Differences in continuous variables in group demographics and t-tau biomarker concentrations were evaluated using either the Mann–Whitney U-test (pilot studies, two groups) or Kruskal–Wallis test with Dunn’s multiple comparison test (clinical cohorts, multiple groups). Fisher’s exact test was used to compare categorical variables between groups (sex). Fold changes for all diagnostic groups were calculated by dividing the t-tau concentration by the mean concentration of the Aβ− control group. The diagnostic accuracy of the measured biomarkers was evaluated using the area under the curve (AUC) from a receiver operating characteristic (ROC) analysis. Statistical differences between the AUC values were determined using the DeLong test. Spearman’s correlation was used to evaluate association of different biomarker concentrations with each other, age and MMSE score. The numbers of biomarker values below the quantification limits or without a read can be found in the Supplementary material and were not included in the analysis. The ROC and correlation analyses included only samples that gave readings with all seven assays to enable reliable comparison. The level of statistical significance was set to *P* < 0.05 (two-tailed).

### Data availability

Blinded data are available upon reasonable request from the corresponding author.

## Results

### Analytical validity of the NTA, NTB and MR total tau assays in CSF

All developed t-tau assays showed appropriate analytical performance. The defined quantification limits for all assays are presented in Supplementary Table 1. The repeatability and intermediate precision of the CSF samples were <30% (results from the validation experiments are presented in Supplementary Table 2 and from the clinical CSF cohorts in Supplementary Table 3), mean spike recoveries were 79–145% (Supplementary Table 4) and recovery percentage with the sample dilution used (1:4) was 78–79% for all t-tau assays (Supplementary Fig. 1).

### Patient demographics

The demographics for the CSF cohorts are presented in Table 1. In the pilot CSF cohort (*n* = 44), the Alzheimer’s disease group was older than the control group (*P* = 0.0015) and included more females (73% versus 32%, *P* = 0.015). No correlation between age and any of the t-tau biomarker levels was present within the diagnostic groups (*r*_s_ = −0.28–0.26, *P* > 0.21 in all).

**Table 1:** Demographics and biomarker concentrations of the CSF cohorts

	Pilot CSF cohort			Clinical CSF cohort 1 (Alzheimer’s continuum)						Clinical CSF cohort 2 (other neurological disorders)			
	Control	AD	*P*	Control	Aβ− MCI	Aβ+ MCI	AD	Non-AD dementia	*P*	CJD	AND	PSP	*P*
*n*	22	22		26	58	33	115	22		24	18	22	
Age, years	71.5 (64.8–75.0)	79.0 (71.0–83.5)	**0.0015**	66.9 (62.7–72.2)*	73.4 (67.8–78.1)^#^	73.7 (67.7–78.1)^#^	73.3 (67.5–78.3)^#^	77.8 (76.1–82.5)*^,#^	**<0.0001**	67.7 (56.1–78.0)	77.2 (63.9–83.6)	68.0 (65.2–75.0)	0.054
Sex, F/M, *n*	7/15	16/6	**0.015**	15/11	30/28	23/10	64/52	11/11	0.50	11/13	11/7	7/15	0.18
MMSE	–	–	**–**	29 (29.0–30.0)*	27 (25.5–28.0)*	26 (24.0–27.0)*	21 (16.0–25.0)	21 (19–23)	**<0.0001**	3.0 (2.0–8.0)	NA	NA	–
CSF Aβ_42_, pg/ml	909 (793–1063)	545 (479–596)	**<0.0001**	1282 (1141–1424)*	1216 (1090–1377)*	592 (483–801)^#^	555 (6491–631)^#^	1096 (919–1198)*	**<0.0001**	851 (742–1219)	894 (640–1303)	947 (756–1138)	0.97
CSF Innotest ® t-tau, pg/ml	324 (228–382)	576 (460–978)	**<0.0001**	227 (181–267)*	281 (209–335)*	316 (291–380)*	777 (610–1020)^#^	254 (215–323)*	**<0.0001**	6579 (2545–11596)^§^	2169 (1701–2389)^§^	208 (177–278)	**<0.0001**
CSF p-tau181, pg/ml	53.0 (41.8–60.3)	80.0 (67.8–118)	**<0.0001**	41.0 (30.0–47.3)*	46.0 (36.8–52.3)*	51.0 (47.5–55.5)*	103 (85.0–133)^#^	38.0 (32.8–44.3)*	**<0.0001**	57.0 (43.8–78.3)§	58.0 (47.0–68.5)^§^	34.5 (28.0–42.8)	**<0.0001**
CSF MR t-tau, pg/ml	210 (116–234)	310 (268–525)	**<0.0001**	114 (76.0–157)*	118 (87.1–170)*	149 (130–169)*	412 (278–587)^#^	131 (74.4–153)*	**<0.0001**	5409 (2691–11210)^§^	1376 (949–2585)^§^	76.1 (54.2–131)	**<0.0001**
CSF NTA t-tau, pg/ml	6.80 (2.90–7.80)	13.8 (9.96–24.9)	**<0.0001**	1.83 (1.26–2.68)*	2.24 (1.05–3.40)*	8.80 (6.04–10.4)^#^	9.95 (5.9–16.2)^#^	1.43 (0.56–3.67)*	**<0.0001**	149 (84.6–268)^§^	120 (65.0–222)^§^	2.04 (0.82–2.84)	**<0.0001**
CSF NTB t-tau, pg/ml	83.9 (49.5–97.4)	142 (86.1–222)	**0.001**	10.8 (7.26–17.7)*	11.1 (5.77–19.4)*	34.6 (24.7–44.6)^#^	47.7 (22.9–72.2)^#^	8.38 (33.4)*	**<0.0001**	2076 (880–5309)^§^	959 (519–2127)^§^	9.59 (5.84–16.4)	**<0.0001**

Data are presented median (interquartile range). Differences between groups were tested with Mann–Whitney U-test (discovery cohort) and Kruskall Wallis test with Dunn’s multiple comparison (clinical cohorts) for continuous variables. Fisher’s exact test was used for categorical variables (sex). *P*-value presents overall difference between groups. Significant differences in pairwise comparisons to *Alzheimer’s disease (AD), ^#^controls and ^§^progressive supranuclear palsy (PSP) groups are also presented. Aβ− = amyloid negative; Aβ+ = amyloid positive; AND = acute neuronal disorders; F = female; M = male.

In clinical CSF cohort 1 (*n* = 228), the controls were younger than the other diagnostic groups (*P* ≤ 0.012), and the other dementia patients older than the Alzheimer’s disease patients (*P* = 0.019). There were no sex differences between the groups (*P* = 0.50). No correlation between age and any of the t-tau biomarker levels was present within the diagnostic groups (*r*_s_ = −0.10–0.31, *P* > 0.06 in all). The MMSE scores decreased gradually from controls [29 (29–30)] to Alzheimer’s disease participants [21 (16–25)].

In clinical cohort 2, the progressive supranuclear palsy and acute neurological disorders groups had normal levels of CSF Aβ_1-42._ CSF t-tau and p-tau levels were significantly higher in CJD and acute neurological disorders versus progressive supranuclear palsy (*P* < 0.0001).

The demographics for the plasma cohorts are presented in Table 2. The Alzheimer’s disease group was older (*P* = 0.0015) and included more females (73% versus 32%, *P* = 0.015) in the pilot cohort for NTB t-tau and Quanterix t-tau. There were no age or sex differences between the other groups. In the clinical plasma cohort, there were no age or sex differences between groups (*P* = 0.71 and *P* = 0.40, respectively). The MMSE scores were significantly higher for the control (*P* = 0.0005) and Aβ-MCI groups (*P* = 0.024) compared with Alzheimer’s disease.

**Table 2:** Demographics and biomarker concentrations of the plasma cohorts

	Plasma pilot cohort (MR and NTA t-tau)			Plasma pilot cohort (NTB and Quanterix t-tau)			Plasma clinical cohort					
	Control	AD	*P*	Control	AD	*P*	Control	Aβ− MCI	Aβ+ MCI	AD	Non-AD dementia	*P*
*n*	20	20		22	22		8	13	6	19	3	
Age, years	69.0 (59.5–77.0)	76.0 (68.3–80.8)	0.077	71.5 (64.8–75.0)	79.0 (71.0–83.5)	**0.0015**	67.0 (54.8–76.5)	66.0 (59.5–77.0)	67.5 (62.8–81.3)	70.0 (65.0–75.0)	71.0 (60.0–76.0)	0.71
Sex, F/M, *n*	9/11	10/10	>0.999	7/15	16/6	**0.015**	4/4	7/6	5/1	15/4	2/1	0.4
MMSE	NA	NA	NA	NA	NA	NA	27.5 (26.3–30.0)*	25.0 (24.0–27.0)*	22.5 (21.8–26.3)	19.0 (13.0–24.0)	24.0 (17.0–28.0)	**0.0006**
CSF Aβ_42_, pg/ml	966 (849)	480 (434–498)	**<0.0001**	909 (793–1063)	545 (479–596)	**<0.0001**	955 (788–1418)*	1032 (617–1108)*	468 (349–680)	547 (450–630)	1571 (730–1821)*	**<0.0001**
CSF t-tau, pg/ml	243 (216–278)	915 (800–978)	**<0.0001**	324 (228–382)	576 (460–978)	**<0.0001**	213 (165–265)*	200 (149–306)*	625 (471–803)	888 (582–1268)	580 (165–1811)	**<0.0001**
CSF p-tau181, pg/ml	42.5 (37.5–45.0)	98.0 (79.5–114)	**<0.0001**	53.0 (41.8–60.3)	80.0 (67.8–118)	**<0.0001**	30.4 (22.0–39.0)*	27.0 (23.6–39.4)*	94.0 (69.7–127)	139 (99.8–204)	49.6 (21.4–62.9)	**<0.0001**
Plasma MR t-tau, pg/ml	44.4 (27.8–64.8)	65.0 (52.9–74.0)	**0.043**	NA	NA	NA	NA	NA	NA	NA	NA	NA
Plasma NTA t-tau, pg/ml	0.034 (0.022–0.053)	0.10 (0.050–0.13)	**0.0056**	NA	NA	NA	0.025 (0.019–0.081)*	0.035 (0.023–0.074)*	0.086 (0.034–0.13)	0.14 (0.095–0.18)	0.041 (0.039–0.074)	**0.0021**
Plasma NTB t-tau, pg/ml	NA	NA	NA	116 (86.9–149)	128 (292.7–167)	0.477	NA	NA	NA	NA	NA	NA
Plasma Quanterix t-tau, pg/ml	NA	NA	NA	1.40 (0.83–1.98)	1.10 (0.98–1.50)	0.55	0.30 (0.18–0.32)	0.27 (0.22–0.34)	0.28 (0.19–0.41)	0.38 (10.30–0.43)	0.28 (0.19–0.38)	0.11

Data are presented median (interquartile range). Differences between groups were tested with Mann–Whitney U-test (pilot cohort) and Kruskall Wallis test with Dunn’s multiple comparison (clinical cohort) for continuous variables. Fisher's exact test was used for categorical variables (sex). *P*-value presents the overall difference between groups. Significant differences in pairwise comparisons to Alzheimer’s disease (AD) are indicated with an asterisk. Quanterix t-tau was measured with Tau 2.0 kit (pilot cohort) and Neurology 3-plex kit (clinical cohort), both assays targeting identical epitopes. Aβ− = amyloid negative; Aβ+ = amyloid positive; F = female; M = male.

### CSF total tau biomarkers across Alzheimer’s disease continuum

**Figure 2:**
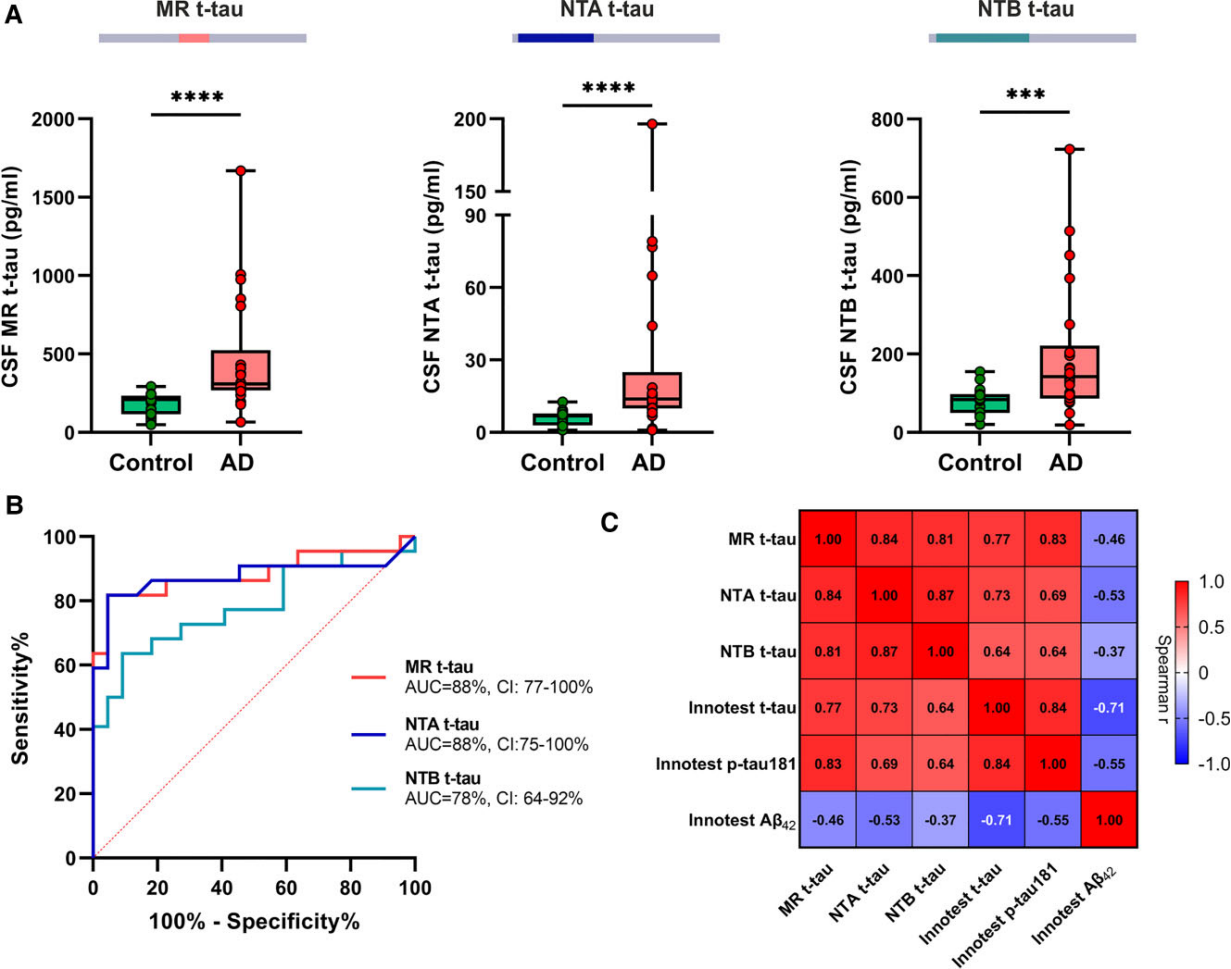
**CSF t-tau biomarker concentrations and their diagnostic performance in Alzheimer’s disease.** (**A**) Box plots presenting in-house MR, NTA and NTB t-tau concentrations in the pilot cohort composed of core CSF biomarker-positive Alzheimer’s disease (AD) and biomarker-negative control patients. (**B**) AUC with 95% CI from ROC analysis showing the diagnostic accuracy of the in-house t-tau assays to distinguish the groups. (**C**) Correlation matrix presenting Spearman’s correlations for all measured t-tau biomarkers with each other and with CSF Aβ_1-42_ in the whole cohort. *P < 0.05; **P < 0.01; ***P < 0.001; ****P < 0.0001; ns = non-significant.

In the pilot cohort, all CSF t-tau biomarkers were significantly increased in Alzheimer’s disease participants versus controls (*P* ≤ 0.001; Fig. 2A). Median fold changes were similar in Alzheimer’s disease [2.3 (1.7–4.2) for NTA, 1.8 (1.1–2.0) for NTB and 1.7 (1.5–2.9) for MR t-tau, *P* = 0.22] and all t-tau assays discriminated Alzheimer’s disease from control patients (AUCs 78%–88%; Fig. 2B). The ROC analysis showed significantly higher AUCs for MR-t-tau [AUC (95% confidence interval): 88% (77–100%), DeLong_MR-NTB_*P* = 0.038] and NTA t-tau [AUC 88% (75–100%), DeLong_NTA-NTB_*P* = 0.043] compared with NTB t-tau [AUC 78% (64–92%)]. All in-house CSF t-tau biomarkers were significantly correlated with each other (*r_s_* ≥ 0.81, *P* < 0.0001 for all) both in the whole cohort (Fig. 2C) and with Innotest t-tau within the diagnostic groups (*r_s_* ≥ 0.44, *P* < 0.040 for all; Supplementary Fig. 2).

**Figure 3:**
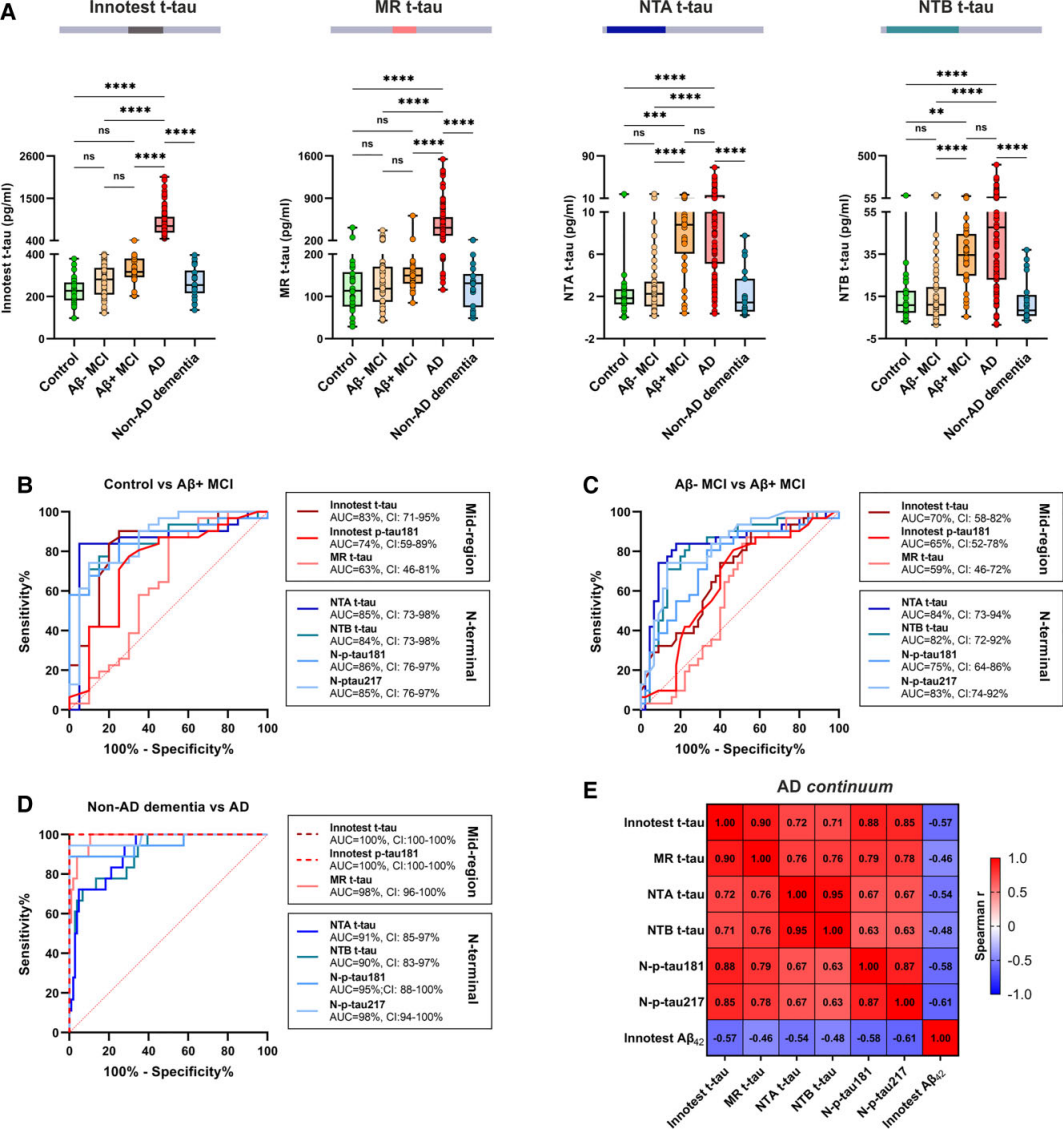
**CSF t-tau biomarker concentrations and their diagnostic performance across the Alzheimer’s disease continuum.** (**A**) Box plots presenting CSF concentrations of Innotest t-tau, in-house MR, NTA and NTB t-tau in clinical cohort 1, including subjects across the Alzheimer’s disease continuum. (**B**) AUC with 95% CI from ROC analysis showing the diagnostic accuracies of all studied CSF biomarkers to distinguish between neurological control and amyloid-positive (Aβ+) cases of MCI; (**C**) amyloid-negative (Aβ−) and Aβ+ cases of MCI; and (**D**) Alzheimer’s disease (AD) and non-Alzheimer’s disease dementia cases (including alcohol-related dementia, vascular dementia, mixed dementia and unspecified dementia). (**E**) Correlation matrix presenting Spearman’s correlations for all measured t-tau and p-tau assays with each other and with CSF Aβ_1-42_ in the whole cohort. *P < 0.05; **P < 0.01; ***P < 0.001; ****P < 0.0001; ns = non-significant.

Results from the clinical CSF cohort 1 agreed with the pilot CSF cohort; all CSF t-tau biomarkers were significantly increased in Alzheimer’s disease compared to controls, Aβ− MCI and other dementias (Fig. 3A, *P* < 0.0001 for all comparisons). For Innotest or MR t-tau, no significant differences between Aβ− MCI and Aβ+ MCI (*P* = 0.58 and *P* > 0.99, respectively) or Aβ+ MCI and controls (*P* = 0.06 and *P* > 0.99, respectively) were observed. On the contrary, both NTA and NTB t-tau showed significantly higher concentrations in Aβ+ MCI in comparison with the controls (*P* = 0.0006 for NTA; *P* = 0.0013 for NTB) and Aβ − MCI (*P* < 0.0001 for both; Fig. 3A).

The ROC curve analysis verified that when differentiating controls from Aβ+ MCI, NTA and NTB t-tau assays performed statistically similarly to all p-tau assays and Innotest t-tau (AUCs 74–86%, DeLong *P* > 0.53 for all comparisons), and significantly better than MR t-tau [AUC 63% (46–81%); DeLong_MR-NTA_*P* = 0.014; DeLong_MR-NTB_*P* = 0.0088; Fig. 3B]. In addition, both NTA and NTB t-tau accurately distinguished Aβ − MCI from Aβ+ MCI [AUC_NTA_ 84% (73–94%), AUC_NTB_ 82% (72–92%)] and exhibited similar performances to N-p-tau217 [AUC 83% (74–92%); DeLong_Np217-NTA_*P* = 0.93; DeLong_Np217-NTB_*P* = 0.88] and N-p-tau181 [AUC 75% (64–86%); DeLong_Np181-NTA_*P* = 0.20; DeLong_Np181-NTB_*P* = 0.30]. In addition, NTA and NTB t-tau assays performed significantly better than MR t-tau [AUC 59% (46–72%); DeLong_MR-NTA,_*P* = 0.0015; DeLong_MR-NTB_, *P* = 0.0012], Innotest t-tau [AUC 70% (58–82%); DeLong_Inno t-tau-NTA,_*P* = 0.046; DeLong_Inno t-tau-NTB_, *P* = 0.056] and Innotest p-tau181 [AUC 65% (52–78%); DeLong_Inno p-181-NTA,_*P* = 0.016; DeLong_Inno p181-NTB_, *P* = 0.024; Fig. 3C] in the same scenario. On the contrary, MR t-tau showed nearly perfect cross-diagnostic performance in discriminating Alzheimer’s disease from other dementias with significantly higher accuracy [AUC 98% (96–100%)] in comparison to NTA and NTB t-tau (AUCs 90–91%, DeLong_MR-NTA,_*P* = 0.013; DeLong_MR-NTA_, *P* = 0.012; Fig. 3D). For this comparison, N-t-tau assays had the same accuracy as N-p-tau assays (DeLong, *P* > 0.06 for all comparisons). Innotest t-tau and p-tau181 were used to stratify patients into diagnostic groups (hence the expected perfect differentiation marked with the dashed line in Fig. 3D).

In clinical CSF cohort 1, all measured CSF tau-species exhibited strong and positive correlation with each other (*r_s_* ≥ 0.63, *P* < 0.0001 for all; Fig. 3E). As expected, strong correlations existed between NTA and NTB t-tau (*r_s_* = 0.95, *P* < 0.0001) and MR and Innotest t-tau (*r_s_* = 0.90, *P* < 0.0001). Each t-tau biomarker showed moderate negative correlation with CSF Aβ_1-42_ in the whole cohort (*r_s_* ≥ −0.46, *P* < 0.0001 for all correlations), but no correlation was observed within any of the diagnostic groups (Supplementary Fig. 3). All new t-tau biomarkers had moderate negative correlation with the MMSE score (*r_s_* ≤ −0.53, *P* < 0.0001 for all correlations; Supplementary Fig. 4).

We also performed an exploratory analysis within the non-Alzheimer’s disease dementia group. Even though our sample sizes were small, NTB t-tau was seen to be significantly lower in vascular dementia (*P* = 0.042), and MR t-tau and Innotest t-tau in mixed dementia (*P*_MR_ = 0.014, *P*_Innotest t-tau_ = 0.022) compared to unspecified dementia. All results are presented in Supplementary Table 5.

### CSF total tau biomarkers in Creutzfeldt–Jakob disease and acute neurological disorders

**Figure 4:**
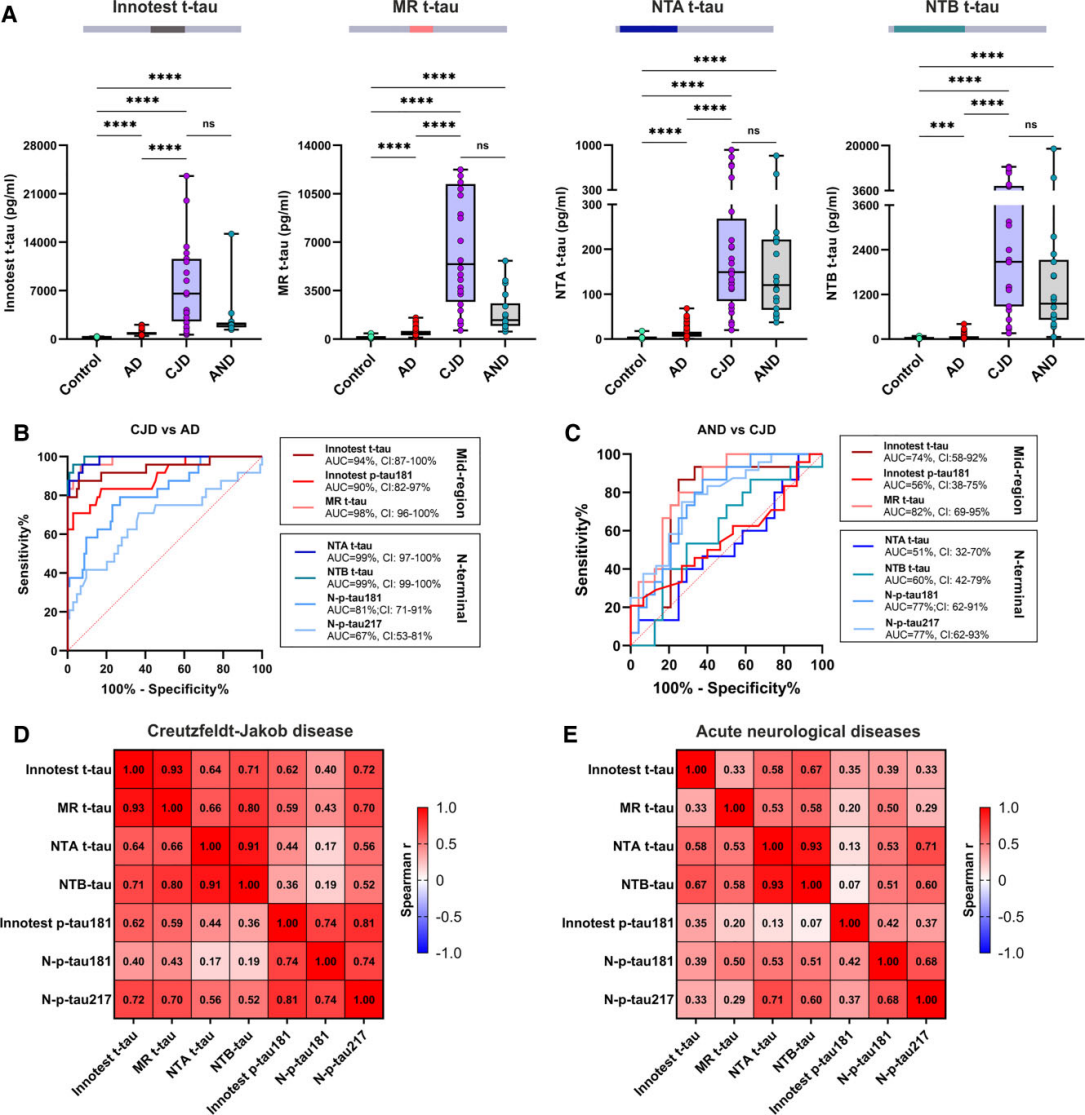
**CSF t-tau biomarker concentrations and their diagnostic performance in CJD and acute neurological disorders.** (**A**) Box plots presenting CSF concentrations of Innotest t-tau, in-house MR, NTA and NTB t-tau in CJD and acute neurological disorders (AND, including individuals with status epilepticus, ischaemic stroke, hepatic encephalopathy and limbic encephalitis). (**B**) AUC with 95% CI from ROC analysis showing the diagnostic accuracies of the tau biomarkers to distinguish between CJD and Alzheimer’s disease (AD) or (**C**) acute neurological disorders. (**D**) Correlation matrix showing Spearman’s correlations for all measured t-tau and p-tau concentrations in CJD and (**E**) acute neurological disorders. *P < 0.05; **P < 0.01; ***P < 0.001; ****P < 0.0001; ns = non-significant.

All t-tau biomarkers were significantly higher in CJD and acute neurological disorders when compared with controls or Alzheimer’s disease (*P* < 0.0001 for all comparisons, Fig. 4A). Median fold changes versus controls were significantly higher for NTA and NTB t-tau compared with MR and Innotest t-tau (Supplementary Table 6); for CJD, the median fold changes were 42 (21–89) for MR t-tau, 57 (32–102) for NTA and 133 (56–341) for NTB t-tau. Similar differences were found for acute neurological disorders, where the mean fold changes were 11 (7.5–20) for MR t-tau, 45 (25–84) for NTA and 61 (33–136) for NTB t-tau. Both NTA and NTB t-tau differentiated CJD from Alzheimer’s disease with an AUC of 99%, performing similarly to the mid-region assays (AUCs 90–98%; DeLong *P* > 0.089 for all comparisons) but significantly better that both N-p-tau assays (AUCs 67–81%; DeLong_NTA-Np217,_*P* < 0.0001; DeLong_NTA-Np181_, *P* = 0.0006; DeLong_NTB-Np217,_*P* < 0.0001; DeLong_NTB-Np181_, *P* = 0.0003; Fig. 4B). Interestingly, the Innotest t-tau, MR t-tau and N-p-tau assays performed better in distinguishing acute neurological disorders and CJD compared with MR p-tau181, NTA and NTB t-tau (Fig. 4C). In both groups, the concentrations of all CSF t-tau assays were positively correlated, with the strongest association between NTA and NTB t-tau (*r*_s_ = 0.91, *P* < 0.0001 in CJD; Fig. 4D; *r_s_* = 0.93, *P* < 0.00001 in acute neurological disorders; Fig. 4E).

An exploratory analysis within the acute neurological disorders group showed no differences in tau concentrations between ischaemic stroke and status epilepticus, whereas all CSF t-tau biomarkers were significantly higher after ischaemic stroke compared to Alzheimer’s disease (*P* < 0.01 for all biomarkers). Hepatic encephalopathy and limbic encephalitis were not included in this analysis (*n* = 1 for both). All results are presented in Supplementary Table 5.

### CSF total tau biomarkers in progressive supranuclear palsy

**Figure 5:**
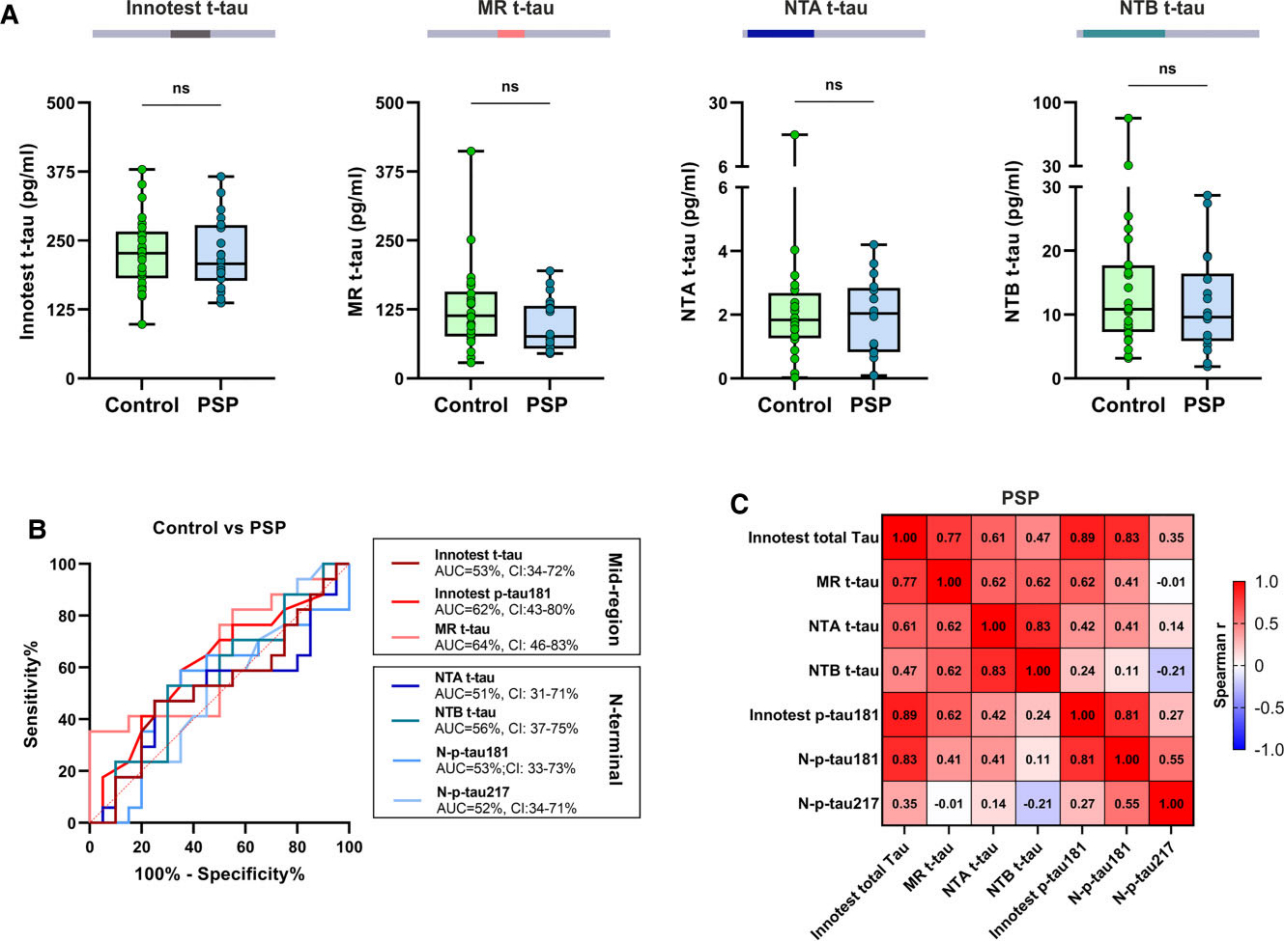
**CSF t-tau biomarker concentrations and their diagnostic performance in progressive supranuclear palsy.** (**A**) Box plots presenting CSF concentrations of Innotest t-tau, in-house MR, NTA and NTB t-tau in progressive supranuclear palsy (PSP). (**B**) AUC from ROC analysis presenting diagnostic accuracies of the tau biomarkers to distinguish between progressive supranuclear palsy and controls. (**C**) Correlation matrix showing Spearman’s correlations for all measured t-tau and p-tau concentrations in progressive supranuclear palsy. *P < 0.05; **P < 0.01; ***P < 0.001; ****P < 0.0001; ns = non-significant.

All CSF t-tau biomarkers were low in progressive supranuclear palsy, with concentrations being similar to the controls (*P* > 0.12 for all assays; Fig. 5A). The median fold changes versus controls were similar and <1 for all t-tau assays (*P* = 0.076, Supplementary Table 6), and none of the t-tau and p-tau assays discriminated progressive supranuclear palsy from controls (AUCs 51–64% for all; Fig. 5B). NTA and NTB t-tau also displayed very strong positive correlations with each other in progressive supranuclear palsy (*r_s_* = 0.83, *P* < 0.0001), and moderate to strong positive correlations with Innotest and MR t-tau (*r_s_* = 0.47−0.62, *P* ≤ 0.059; Fig. 5C). There was no association between the N-t-tau and N-p-tau concentrations in progressive supranuclear palsy (*r*_s_ = −0.21–0.41, *P* ≥ 0.10).

### Plasma total tau biomarkers in Alzheimer’s disease

**Figure 6:**
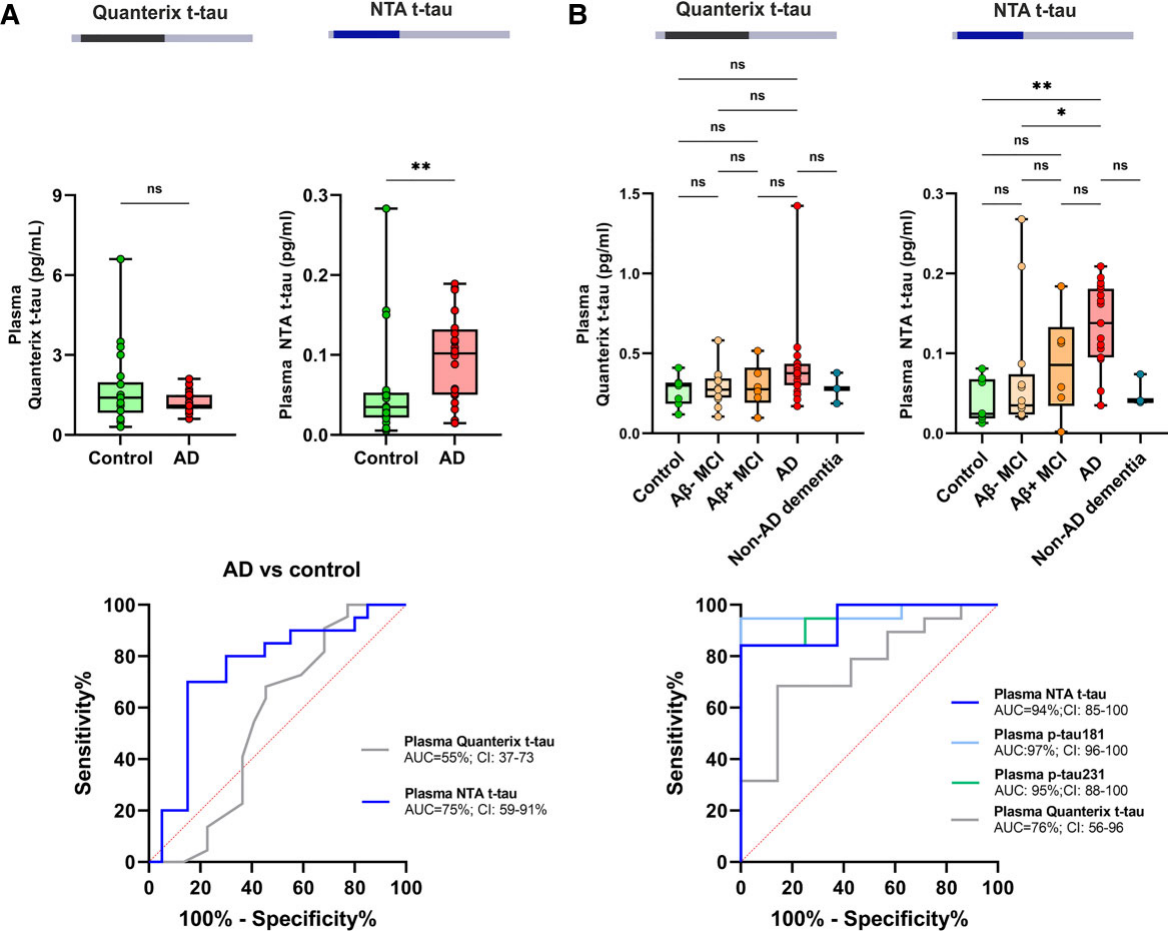
**Plasma NTA and Quanterix t-tau concentrations and its diagnostic performance in Alzheimer’s disease.** Box plots presenting plasma Quanterix t-tau and NTA t-tau concentrations and AUC from ROC analysis (**A**) in the pilot cohort composed of Alzheimer’s disease (AD) and control patients, and (**B**) in a clinical cohort including subjects with Alzheimer’s disease, amyloid-negative (Aβ−) and amyloid-positive (Aβ+) MCI, controls and non-Alzheimer’s disease dementia. *P < 0.05; **P < 0.01; ***P < 0.001; ****P < 0.0001; ns = non-significant.

In the pilot plasma cohort, the NTA t-tau levels showed significantly higher concentrations in Alzheimer’s disease than in control patients (*P* = 0.0056) and the clearest differentiation between the two groups, with an AUC of 75% (59–91%) (Fig. 6A). More overlap but a statistically significant difference between the groups was also seen in the MR t-tau levels (*P* = 0.043), whereas Quanterix Tau 2.0 and NTB t-tau showed similar concentrations in both groups (*P* = 0.55 and *P* = 0.48, respectively; Supplementary Fig. 5). However, in this small cohort, the plasma t-tau biomarker concentrations did not correlate with CSF Innotest t-tau (*r_s_* = 0.29, *P* = 0.069 for MR t-tau; *r_s_* = 0.29, *P* = 0.071 for NTA t-tau; *r_s_* = 0.15, *P* = 0.35 for NTB t-tau; Supplementary Fig. 6).

Based on the pilot results, we aimed to replicate the promising findings with NTA in another clinical cohort comprising patients across the Alzheimer’s disease continuum. Again, higher plasma NTA t-tau concentrations were measured in patients with Alzheimer’s disease than in controls (*P* = 0.0033) or patients with Aβ − MCI (*P* = 0.027), whereas no statistically significant differences were observed with the Quanterix t-tau (*P* = 0.44 and 0.23, respectively; Fig. 6B). Plasma NTA t-tau differentiated Alzheimer’s disease patients and controls with an AUC of 94% (85–100%), performing similarly to plasma N-p-tau181 [AUC 97% (96–100%)] and N-p-tau231 [AUC 95% (88–100%)] and better than Quanterix plasma t-tau [AUC 76% (56–96%)]. Plasma NTA t-tau also showed strong correlation with CSF t-tau (*r_s_* = 0.61, *P* > 0.0001), plasma N-p-tau181 (*r_s_* = 0.68, *P* < 0.0001) and plasma N-p-tau231 (*r_s_* = 0.69, *P* < 0.0001) in the whole cohort, whereas no correlation with Quanterix plasma t-tau was observed (*r_s_* = 0.22, *P* = 0.13) (Supplementary Fig. 7).

## Discussion

Based on the current understanding about the complexity of tau protein, it has become obvious that it is a much more challenging biomarker to interpret than previously thought.^11^ In brain, tau is mostly present as a full-length protein, whereas many different fragments of diverse lengths are known to exist in CSF, and these increase in concentration during the pathological process of Alzheimer’s disease.^17–19,33–35^ Studies using immunoprecipitation followed by mass spectrometry have shown that tau peptides C-terminal to position 254 are not detectable in CSF or blood, suggesting that tau content in these fluids consists of tau x-254 forms.^19,22,24,26^ However, extracting conclusions about the biomarker potential of different fragments is difficult, since no direct comparison between assays targeting mid-region and N-terminal phosphorylated and non-phosphorylated species has been performed on the same analytical platform or in the same large clinical cohorts. Since N-terminal tau forms are ubiquitous to CSF and blood, targeting this part of the tau molecule could generate biomarkers applicable to both fluid systems. We took advantage of our recent successful development of N-terminal-directed p-tau181, p-tau217 and p-tau231 biomarkers^20,21,24–26,32^ to design two novel ultrasensitive t-tau immunoassays (NTA and NTB t-tau) targeting N-terminal epitopes. To enable cross-biomarker comparisons using an identical analytical technology, we developed a third assay (MR t-tau) using the same antibodies used in the gold-standard Innotest t-tau. Thereafter, we performed diagnostic comparisons between the new versus classical t-tau biomarkers (with N-p-tau assays included for proof of concept) across the Alzheimer’s disease continuum and other neurological disorders using the same Simoa platform.

### N-terminal bearing CSF tau biomarkers in Alzheimer’s disease

Consistent with previous findings in CSF,^17–19,35^ we showed that all measured CSF mid-region and N-terminal t-tau fragments were increased in Alzheimer’s disease compared with controls and had high diagnostic accuracies for differentiating the two groups (AUCs 90–98% for all assays). However, in our study, only levels of N-terminal-bearing CSF tau biomarkers measured by NTA (HT7/Tau12) and NTB (1−100/BT2) t-tau increased significantly in early Aβ+ MCI when compared with controls. In addition, only NTA and NTB t-tau were able to distinguish between Aβ+ MCI and Aβ− MCI, showing equal performances as N-p-tau217 and N-p-tau181. On the contrary, all t-tau assays measured in CSF distinguished Alzheimer’s disease and other dementia cases with high accuracy (AUCs 90–100%). In this context, CSF MR t-tau performed significantly better than NTA or NTB (DeLong *P* ≤ 0.0012), but there were no significant differences between the CSF N-t-tau and N-p-tau assays (Delong *P* ≥ 0.06). Together, our findings agree with the previously presented hypothesis that during the Alzheimer’s disease pathophysiological process, tau fragments including the N-terminus are released early by neurons that are presumably affected by Aβ toxicity but still only at risk of developing tangle pathology.^11,22^ We also showed that this early increase can be detected with N-terminal directed assays targeting both phosphorylated and non-phosphorylated epitopes.

CSF t-tau is an established core Alzheimer’s disease biomarker, and previous studies have shown increased concentrations measured with classical mid-region assays at the MCI stage of Alzheimer’s disease.^2,36,37^ However, in our study, mid-region-bearing fragments were still not significantly different in MCI (both Aβ− and Aβ+) than in controls. These seemingly contradictory findings could be explained by the fact that earlier Aβ+ MCI (A+/T−/N−) patients were included in our CSF study, and conversely, significant increases only in N-terminal-bearing tau biomarkers could be observed at this stage (Fig. 3A). This is in agreement with previous findings using p-tau assays in the same cohort, namely that CSF mid-p-tau181 likely reflects more established tau pathology in Alzheimer’s disease, whereas abnormal levels of N-p-tau181 or N-p-tau217 in early MCI were suggested to have a closer association with initial Aβ changes.^20^

The developed NTA and NTB t-tau assays use mid-region-targeted antibodies for capture (HT7 and BT2), meaning that the mid-region t-tau assays should also be able to capture the shorter N-terminal species bearing these epitopes. However, in our study, this was not the case. This apparent discrepancy might be explained by differences in the quantity of the different fragments. Using the same recombinant tau as the assay calibrator, we observed that the levels of CSF t-tau measured by NTA (0–85 pg/ml) and NTB (0–480 pg/ml) assays were pronouncedly lower than those measured by MR t-tau (0–2200 pg/ml) and Innotest t-tau (0–2600 pg/ml). Furthermore, tau truncation and excretion are regulated processes, and it has been hypothesized that, whereas full length tau is passively secreted from neurons, truncated forms are released through active secretion.^38^ Thus, the subtle but meaningful changes in the levels of N-terminal fragments could be diluted by the excess of other, longer tau fragments in CSF, captured by mid-region t-tau assays.

### CSF tau biomarkers in non-Alzheimer’s disease dementia

In addition to exploring different stages within the Alzheimer’s continuum, it would be interesting to investigate differences in various CSF t-tau markers between different non-Alzheimer’s disease dementias. Here, we reported that all tau biomarkers were significantly higher in Alzheimer’s disease in comparison to other dementias and some differences were also observed within the non-Alzheimer’s disease dementia group; NTB t-tau was significantly lower in vascular dementia, and all mid-region fragments (MR t-tau, Innotest t-tau and Innotest p-tau181) in mixed dementia in comparison to unspecified dementia. However, due to our small sample size (3–8 per group) and heterogeneous nature of different dementias, we want to emphasize that these findings should be interpreted with caution and investigated in more detail on a larger non-Alzheimer’s disease dementia cohort in the future.

### N-terminal bearing CSF tau biomarkers in other neurological diseases

In addition to Alzheimer’s disease, CSF t-tau is known to be highly increased in CJD and acute neurological disorders due to the occurrence of rapid and aggressive neurodegeneration in these diseases. As expected, we also observed highly elevated concentrations of all investigated CSF t-tau biomarkers in both groups. Fold changes calculated against controls were higher for the N-t-tau biomarkers in comparison to MR t-tau, and measuring the N-terminal fragments improved the diagnostic accuracy between Alzheimer’s disease and CJD. However, the groups were well distinguished by all CSF t-tau biomarkers, thus this difference is likely not clinically relevant. In our study, we did not see differences between the CSF mid-region or N-terminal assays in their ability to differentiate non-Alzheimer’s disease dementia or progressive supranuclear palsy from controls. Previously, abnormally low levels of CSF t-tau were reported in progressive supranuclear palsy using in-house ELISAs targeting the same N-terminal epitopes as our in-house NTA and NTB t-tau assays for Simoa.^3^ Our results support the present understanding that tau deposition and metabolism in primary tauopathies differ from that in Alzheimer’s disease, and other forms of tau with better biomarker potential should still be explored to address these disorders.

### Short N-terminal-bearing tau fragments as plasma biomarkers in Alzheimer’s disease

Due to less invasive sampling and higher cost-effectiveness, blood biomarkers hold enormous potential for the screening and diagnosis of Alzheimer’s disease when compared to both CSF and imaging biomarkers. Plasma t-tau has also been shown to be increased in Alzheimer’s disease when compared to controls and Aβ+ MCI, however, high overlap between the diagnostic groups and lack of correlation with CSF t-tau has hindered its usability.^28,29^ This could be due to the interference caused by peripheral expression of tau and/or rapid metabolism and fragmentation of tau in plasma, resulting in fragments that might not be recognized by the commercial t-tau assays. Recently, plasma NT1, targeting shorter, N-terminal-bearing fragments of tau (BT2/Tau12), was able to differentiate Alzheimer’s disease from control subjects and predict future cognitive decline, suggesting that N-t-tau fragments could be more suitable blood biomarkers.^18,31,39^ In this study, we identified a novel plasma N-terminal biomarker (NTA) that also showed higher concentrations in Alzheimer’s disease compared with controls both in the pilot and clinical cohorts. Interestingly, plasma NTA concentrations in the clinical cohort also correlated strongly with CSF t-tau, as well as both plasma p-tau181 and p-tau231. These findings agree with previous studies suggesting that short, N-terminal-bearing fragments in plasma may present the same early response to Aβ seen in CSF and be less prone to degradation than the longer fragments.^18,31^

In contrast to CSF, where our NTA t-tau showed similar performance to NTB t-tau, the NTA t-tau assay (requiring a short minimum aa sequence of 6–159) performed better in plasma compared with NTB t-tau (requiring a longer minimum aa sequence of 6–198, similar to NT1^18^). Previously, Chen *et al.*^18^ also showed that NT1 performed better than another N-terminal biomarker NT2 (ADx202/Tau12) that requires a longer sequence (aa 6–224). When we compared our N-terminal assays with the commercial Quanterix t-tau (that could also be considered to target a N-terminal sequence, since it requires a sequence ranging from aa 16–222), we saw poor performance of this biomarker in plasma in two different cohorts compared with both NTA and NTB t-tau. Even though the NTA and NTB t-tau still need to be further optimized and validated for blood, our findings (together with the earlier reports on NT1 and NT2) support the view that assays targeting minimal N-terminal sequences (including aa 6 targeted by the Tau12 and 1–100 antibodies) provide superior performance in detecting Alzheimer’s disease-relevant tau species in plasma when compared with assays targeting longer N-terminal or mid-region fragments.

### Strengths and limitations

A clear strength of this study is the identification of a novel short NTA t-tau biomarker measurable using Simoa technology. Another strength of the present study was the comparison of our in-house MR and N-terminal t-tau biomarkers with both classical CSF tau biomarkers (Innotest t-tau and p-tau181), and previously described in-house N-terminally targeted p-tau biomarkers (N-p-tau181 and N-p-tau217)^20^ in the same memory clinic cohort, translating well into real-world clinical settings. Different assays were also evaluated both in CSF and in plasma. In addition, we developed MR t-tau that mimics the gold standard Innotest t-tau in Simoa, thus we can be sure that analytical platform or technical effects do not influence our comparison. However, our study does not go without limitations. First, due to the cohorts being composed of individuals recruited from a memory clinic setting, our study does not include any samples from an early, preclinical phase of Alzheimer’s disease. Thus, we were unable to investigate how early in the Alzheimer’s disease continuum the N-terminal t-tau fragments become abnormal. Second, due to the cross-sectional nature of this study, we were not able to compare the longitudinal changes of the different biomarkers across the Alzheimer’s disease continuum. In addition, the *APOE* status was not available for all participants in the clinical cohorts, thus we could not investigate the effect of *APOE* on the t-tau biomarker levels in this study.

## Conclusion

In conclusion, we developed new t-tau immunoassays for the Simoa platform targeting both N-terminal and mid-region epitopes of tau and showed that different t-tau assays have different biomarker potential in the Alzheimer’s disease continuum both in CSF and plasma. NTA and NTB t-tau were able to discriminate MCI with and without underlying Aβ pathology, and therefore detecting early Alzheimer’s disease-related abnormalities in CSF, whereas all t-tau assays showed excellent performance in differentiating Alzheimer’s disease from other dementias. In addition, N-terminal-directed CSF t-tau biomarkers were seen to be increased to higher degrees in CJD and acute neurological disorders, both characterized by aggressive neurodegeneration. Most notably, plasma NTA t-tau was able to successfully differentiate Alzheimer’s disease from controls and correlated strongly with both plasma N-p-tau181 and N-p-tau231. Based on our findings, N-terminal-bearing forms of tau seem to be secreted into CSF in an early phase of the Alzheimer’s disease pathological process, and like N-p-tau, N-t-tau biomarkers could provide added value in the variety of tau assays available for further research.

## Supplementary Material

awab481_Supplementary_Data

## Abbreviations

Aβ: amyloid-β
CJD: Creutzfeldt–Jakob disease
MCI: mild cognitive impairment
MR t-tau: total tau assay targeting mid-region-bearing fragments
MMSE: Mini-Mental State Examination
N-p-tau181/tau217: p-tau assay targeting N-terminal-directed tau phosphorylated at threonine-181/217
NTA/B: N-terminal-directed total tau assay A/B
