# Supplementary material for: N-terminal and mid-region tau fragments as fluid biomarkers in neurological diseases
Source: Brain. 2022 Jul 27;145(8):2834–48. doi: 10.1093/brain/awab481 (PMC9420020; doi:10.1093/brain/awab481)
Supplement: awab481_Supplementary_Data [file awab481_supplementary_data.pdf]

Supplementary material

N-terminal and mid-region tau fragments as fluid biomarkers in neurological diseases

Anniina Snellman[1,2*], Juan Lantero-Rodriguez[1*], Andreja Emeršič[3,4], Agathe Vrillon[5,6], Thomas K. Karikari[1,7], Nicholas J. Ashton[1,8,9,10], Milica Gregorič Kramberger[3,11], Saša Čučnik[3,4,12], Claire Paquet[5,6], Uroš Rot[3,11], Henrik Zetterberg[1, 13, 14,15,16] and Kaj Blennow[1,13 #]

[1]Department of Psychiatry and Neurochemistry, Institute of Neuroscience & Physiology, the Sahlgrenska Academy at the University of Gothenburg, Mölndal, Sweden.

[2]Turku PET Centre, University of Turku, Turku, Finland.

[3]Department of Neurology, University Medical Centre Ljubljana, Slovenia.

[4]Faculty of Pharmacy, University of Ljubljana, Slovenia.

[5]Université de Paris, Cognitive Neurology Center, GHU Nord APHP Hospital Lariboisière Fernand Widal, Paris, France.

[6]Université de Paris, Inserm UMR S11-44 Therapeutic Optimization in Neuropsychopharmacology, Paris, France.

[7]Department of Psychiatry, University of Pittsburgh, Pittsburgh, PA, USA.

[8]Wallenberg Centre for Molecular and Translational Medicine, University of Gothenburg, Gothenburg, Sweden.

[9]Department of Old Age Psychiatry, Maurice Wohl Clinical Neuroscience Institute, King's College London, London, UK.

[10]NIHR Biomedical Research Centre for Mental Health & Biomedical Research Unit for Dementia at South London & Maudsley NHS Foundation, London, UK.

[11]Faculty of Medicine, University of Ljubljana, Slovenia.

[12]Department of Rheumatology, University Medical Centre Ljubljana, Slovenia.

[13]Clinical Neurochemistry Laboratory, Sahlgrenska University Hospital, Mölndal, Sweden.

[14]Department of Neurodegenerative Disease, UCL Institute of Neurology, Queen Square, London, UK.

[15]UK Dementia Research Institute at UCL, London, UK.

[16]Hong Kong Center for Neurodegenerative Diseases, Hong Kong, China.

Supplementary methods

T-tau immunoassay development and validation for CSF

For NTA t-tau and MR t-tau, mouse monoclonal antibody targeting aa 159-163 (HT7, #MN1000, Thermo Scientific) and for NTB t-tau mouse monoclonal antibody targeting aa 6-18 (1-100, #816601, BioLegend) were conjugated to magnetic homebrew carboxylated beads (#103207, Quanterix). All detector antibodies were biotinylated in-house with EZ-Link™ NHS-PEG4-Biotin (Thermo Scientific, USA). NTA and MR t-tau were measured using a 2-step protocol, where the assay beads, detector antibody and the analyte are first co-incubated, followed by washing of the beads, and incubation with the enzyme. NTB t-tau was measured using a 3-step protocol, where the beads were initially incubated with the analyte, then with the detector, and finally with the enzyme. After another wash, the substrate resorufin β−D-galactopyranoside (RGP; #103159, Quanterix) was added to the beads to gain the measured fluorescent signal. Recombinant non-phosphorylated full-length Tau-441 (#T08-54N, SignalChem) was used as a calibrator in all the in-house t-tau assays. Three-fold diluted calibrator series in assay diluent (Tau 2.0 diluent, #101556, Quanterix) ranging from 500 to 0.7 pg/ml (MR and NTB t-tau), 56 to 0.1 pg/ml (CSF NTA t-tau) or 18.5 to 0.03 pg/ml (plasma NTA t-tau) were included in each sample plate.

Limits of detection (LOD) and lower limits of quantification (LLOQ) were defined by running 16 blank samples in duplicates and adding three or ten standard deviations to the measured mean value, respectively. Upper limits of quantification (ULOQ) were set as the concentration of the highest calibration accounting for the used dilution factor. For dilution linearity, two human CSF samples (pooled Alzheimer's disease and pooled controls) were measured with 2-fold dilution series (1:2, 1:4, 1:8) as duplicates. For spike recovery, two CSF samples were diluted 1:2 and spiked 10 pg/ml and 40 pg/ml (NTA t-tau) or 50 pg/ml and 150 pg/ml (NTB and MR t-tau) of exogenous non-P Tau441 (the assay calibrator). Spiked samples were then measured on a same plate with non-spiked CSF and spiked assay diluent. Spike recovery was calculated as: % Recovery = Concentration of spiked sample / (Concentration of non-spiked sample + Concentration of spiked buffer). Precision and accuracy were tested by measuring five replicates of two human CSF samples as duplicates in three consecutive days and calculating the intra-assay precision (variation within run, CV_r (%)) and inter-assay precision (variation between runs, CV_{rw} (%)).

Supplementary results

T-tau assay development and validation for CSF

Analytical performance of all the in-house assays were acceptable. Mean spike recovery was 79.3% (range 76-84%) for NTA t-tau, 145% (range 133-157%) for NTB t-tau, and 123% (range 118-126%) for MR t-tau assays (**Supplementary Table 4**).

Recovery % with the used sample dilution (1:4) was 78-79% with all assays (**Supplementary Figure 1**). With dilution, measured concentrations became lower in all in-house assays, implying that the sample matrix has some interference with the obtained signal. However, when comparing samples with the same dilution, this should not be problematic. Due to extremely high t-tau concentrations, some of the Creutzfeldt-Jakob disease and acute neurological disorders samples were further diluted 1:10, and these measurements were above the ULOQ (2000 pg/ml) and all extrapolated from the standard curve. This should be kept in mind and the concentrations gained from these analyses are likely underestimations and should be interpreted with caution.

T-tau measurements for CSF

All pilot CSF cohort samples measured above the quantification limits of each t-tau assay. From the clinical cohorts, 26/292 (8.9%) CSF samples were below the LOD of NTA t-tau (six controls, nine Aβ- subjects with mild cognitive impairment (MCI), one Aβ+ MCI, three non-Alzheimer's disease dementia, four progressive supranuclear palsy, and three Alzheimer's disease), and 5/292 (1.7%) of NTB t-tau (two controls, one Aβ- MCI, one Aβ+ MCI, and one non-Alzheimer's disease dementia). One CSF sample measured with NTB (Aβ- MCI subject) and one sample measured with MR t-tau (Aβ- MCI subject) was excluded due to technical error (no reading). In addition, one CSF sample was finished before measurement with NTB (Aβ- MCI).

All t-tau levels were extremely high in Creutzfeldt-Jakob disease and acute neurological disorders, and most of the samples gave readings above the defined upper limit of quantification (ULOQ) even with the additional 1:10 dilution. Thus, majority of the concentrations were extrapolated from the calibration curve (>2000g/ml) and results should be interpreted with caution.

Supplementary tables

Supplementary Table 1 Quantification limits for NTA, NTB and MR t-tau assays for CSF

	NTA t-tau	NTB t-tau	MR t-tau
**LOD (pg/ml)**	0.098	0.29	0.26
**ULOQ (pg/ml)**	224	2000	2000
**LLOQ (pg/ml)**	0.28	1.0	1.2

Abbreviations: LOD, limit of detection; LLOQ, lower limit of quantification; ULOQ, Upper level of quantification.

Supplementary Table 2 Repeatability and intermediate precision of NTA, NTB and MR t-tau in CSF (results from validation experiments)

	Sample	Conc (pg/ml)	Repeatability (CVr) %	Intermediate precision (CVRw) %
**NTA t-tau**	CSF1	8	1.5	16.9
	CSF2	36	7.2	16.7
	**Mean**		**4.4**	**16.8**
**NTB t-tau**	CSF1	86	8.1	27.4
	CSF2	122	4.1	29.6
	**Mean**		**6.1**	**28.5**
**MR t-tau**	CSF1	244	2.7	9.6
	CSF2	280	3.6	15.3
	**Mean**		**3.2**	**12.5**

Supplementary Table 3 Repeatability and intermediate precision of NTA, NTB and MR t-tau in CSF (results from clinical cohorts 1 and 2)

	Sample	Conc (pg/ml)	Repeatability (CVr) %	Intermediate precision (CVRw) %
**NTA t-tau**	LowQC	9	6.5	15.8
	HighQC	35	4.0	15.0
	**Mean**		**5.3**	**15.4**
**NTB t-tau**	LowQC	50	11	15
	HighQC	67	10	15.5
	**Mean**		**10.5**	**15.3**
**MR t-tau**	LowQC	9	6.8	14.4
	HighQC	263	8.0	15.0
	**Mean**		**7.4**	**14.7**

Supplementary Table 4 Spike recovery of NTA, NTB and MR t-tau in CSF

	Sample		Measured (pg/ml)	Mean (pg/ml)	%CV conc	Expected conc. (pg/mL)	Spike recovery (%)
NTA t-tau	CSF1	Neat	6.69<br>6.70	6.69	0		
		High spike	39.8<br>40.0	39.9	0.2	46.7	**77.5**
		Low spike	10.4<br>11.2	10.8	5.1	16.7	**76.0**
	CSF2	Neat	10.1<br>10.6	10.3	3.5		
		High spike	47.3<br>45.6	46.4	2.7	50.3	**84.2**
		Low spike	14.3<br>14.1	14.2	1.2	20.3	**79.5**
	Diluent	High spike	46.2<br>43.4	44.8	4.5	40	
		Low spike	7.68<br>7.41	7.54	2.6	10	
NTB t-tau	CSF1	Neat	48.0<br>47.4	47.7	0.9		
		High spike	312<br>296	304	3.8	198	**157**
		Low spike	135<br>144	139	4.6	97.7	**133**
	CSF2	Neat	69.6<br>69.8	69.7	0.2		
		High spike	322<br>343	332	4.3	220	**155**
		Low spike	167<br>172	170	2.2	120	**134**
	Diluent	High spike	152<br>139	145	6.2	150	
		Low spike	54.7<br>58.9	56.8	5.3	50	
MR t-tau	CSF1	Neat	226<br>227	226	0.2		
		High spike	474<br>493	483	3.4	376	**126**
		Low spike	316<br>324	320	2.1	276	**118**
	CSF2	Neat	234<br>228	231	1.8		
		High spike	494<br>513	503	2.7	381	**130**
		Low spike	323<br>328	325	1	281	**118**
	Diluent	High spike	316<br>313	157	0.8	150	
		Low spike	109<br>73.8	45.7	27.2	50	

Supplementary Table 5 Innotest, MR, NTA and NTB t-tau levels within the non-Alzheimer's disease dementia and acute neurological disorders groups (clinical cohorts 1 and 2)

	**Non-AD dementia**					**Acute neurological diseases (and AD)**			
	**Alcohol related dementia**	**Vascular dementia**	**Mixed vascular/non-AD cortical dementia**	**Unspecified dementia**	***P***	**Ischemic stroke**	**Status epilepticus**	**AD**	***P***
**N**	3	4	7	8		7	9	112	
**CSF Aβ42 (pg/ml)**	925 (773-1249)	1084 (1029-1302)	1115 (663-1192)	1096 (936-1248)	**0.89**	984 (645-1220)*	773 (578-1287)*	555 (6491-631)	**<0.0001**
**CSF Innotest ® t-tau (pg/ml)**	292 (220-362)	254 (185-299)	201 (161-236)#	322 (256-373)	**0.03**	2270 (2039-2500)*	1972 (1446-2326)*	777 (610-1020)	**<0.0001**
**CSF p-tau181 (pg/ml)**	34.0 (31.0-45.0)	41.5 (31.0-46.0)	33.0 (31.0-34.0)#	43.5 (57.5-49.0)	**0.046**	61.0 (56.0-83.0)*	48.0 (43.5-66.0)*	103 (85.0-133)	**<0.0001**
**CSF MR t-tau (pg/ml)**	137 (131-165)	123 (65.2-135)	77.0 (60.0-117)#	163 (132-202)	**0.0098**	1156 (881-3219)*	1676 (1234-2906)*	412 (278-587)	**<0.0001**
**CSF NTA t-tau (pg/ml)**	0.73 (0.23-1.77)	0.87 (0.61-1.32)	1.48 (0.64-4.9)	2.8 (0.69-5.8)	**0.37**	112 (67.9-834)*	128 (69.0-207)*	9.95 (5.9-16.2)	**<0.0001**
**CSF NTB t-tau (pg/ml)**	6.3 (4.7-11.6)	5.68 (5.4-6.1)#	8.5 (4.5-16.8)	15.6 (8.8-31.8)	**0.033**	647 (558-8265)*	1050 (498-1881)*	47.7 (22.9-72.2)	**<0.0001**

Data presents as median (interquartile range). Significant differences in pairwise comparisons to unspecified dementia (#) and AD (*) are presented. Hepatic encephalopathy and limbic encephalitis were not included in the analysis (n = 1 for both).

Supplementary Table 6 Fold changes to controls for Innotest, MR, NTA and NTB t-tau (clinical cohorts 1 and 2)

	Innotest	MR t-tau	NTA t-tau	NTB t-tau	*P*
**Aβ- control**	1.0 (0.8-1.2)	0.9 (0.6-1.2)	0.7 (0.5-1.0)	0.7 (0.5-1.1)	0.11
**Aβ- MCI**	1.2 (0.9-1.4)	0.9 (0.7-1.3)	0.9 (0.4-1.3)*	0.7 (0.4-1.2)*	**0.0004**
**Aβ+ MCI**	1.4 (1.3-1.7)	1.2 (1.0-1.3)	3.3 (2.3-4.0)*	2.2 (1.6-2.9)*	**< 0.0001**
**NADD**	1.1 (0.9-1.4)	1.0 (0.6-1.2)	0.5 (0.2-1.4)*	0.5 (0.4-1.0)*	**0.0048**
**AD**	3.4 (2.7-4.5)	3.3 (2.2-4.7)	3.7 (1.9-6.2)	3.1 (1.5-4.6)	0.064
**CJD**	29 (11-51)	42 (21-89)	57 (32-102)*, #	133 (56-341)*, #	**< 0.0001**
**AND**	9.5 (7.4-10)	11 (7.5-20)	45 (25-84)*, #	61 (33-136)*, #	**< 0.0001**
**PSP**	0.9 (0.8-1.2)	0.6 (0.4-1.0)	0.8 (0.3-1.1)	0.6 (0.4-1.1)	0.076

Data presents as median (interquartile range). Significant differences in pairwise comparisons to Innotest t-tau (*) and MR t-tau (#) are presented.

Supplementary figures

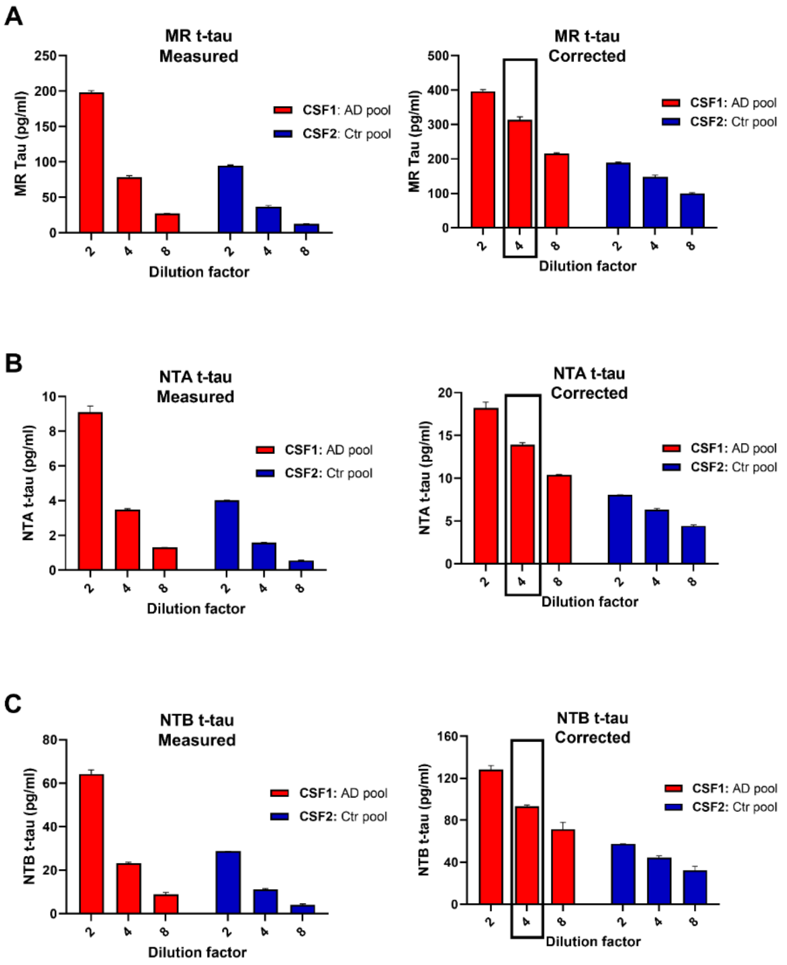

Supplementary Figure 1 Dilution linearity of MR, NTA, and NTB t-tau in CSF

CSF pooled from different Alzheimer's disease (CSF1: AD pool) or control (CSF2: Ctr pool) samples were measured diluted two-, four- and eight-fold. Measured concentrations and concentrations corrected for dilution factors are presented for **(A)** MR t-tau, **(B)** NTA t-tau and **(C)** NTB- t-tau. Sample dilution used for further CSF analysis (1:4) is highlighted in the figure.

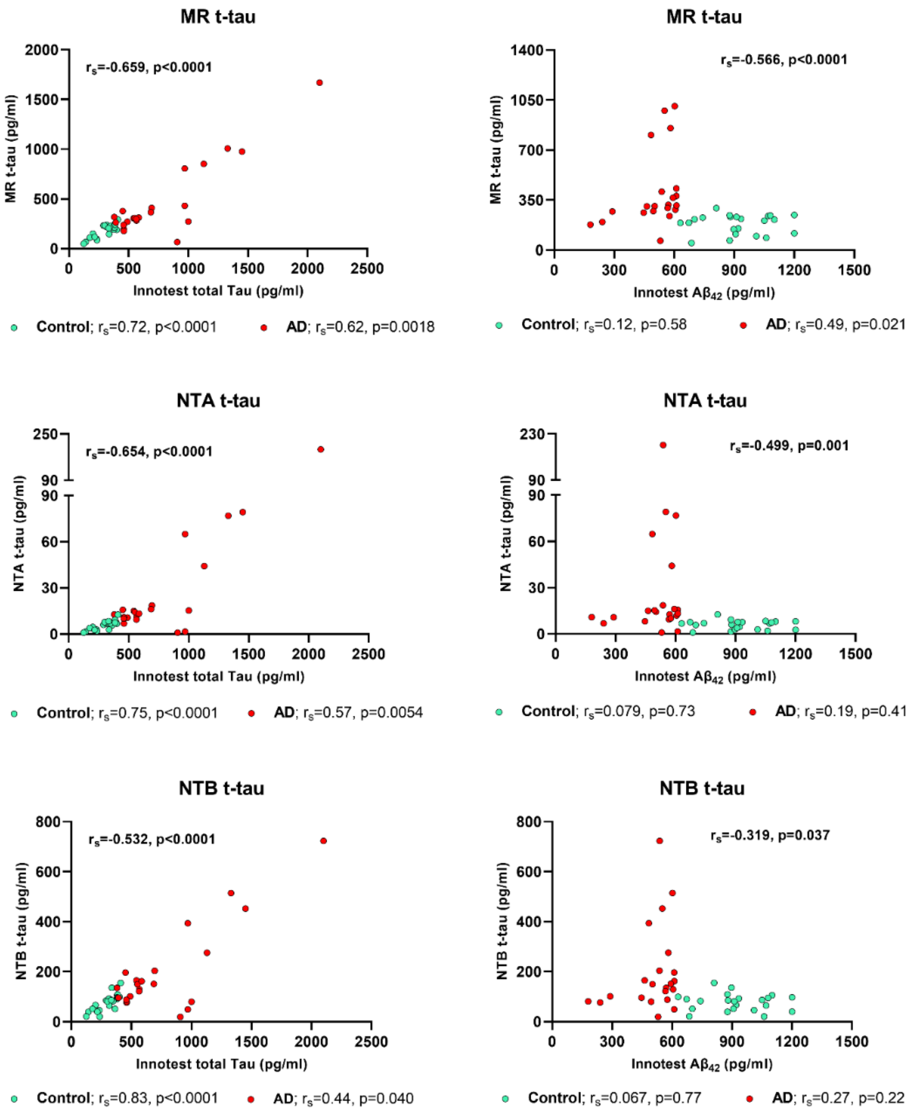

Supplementary Figure 2 Correlation of CSF MR, NTA, and NTB t-tau with Innotest CSF t-tau and CSF β-amyloid$_{1\text{-}42}$ (CSF pilot cohort).

Association between developed in-house CSF t-tau biomarkers with Innotest t-tau and Innotest amyloidβ$_{42}$ in the CSF pilot cohort was evaluated using Spearman's correlation both in the whole cohort and within diagnostic groups.

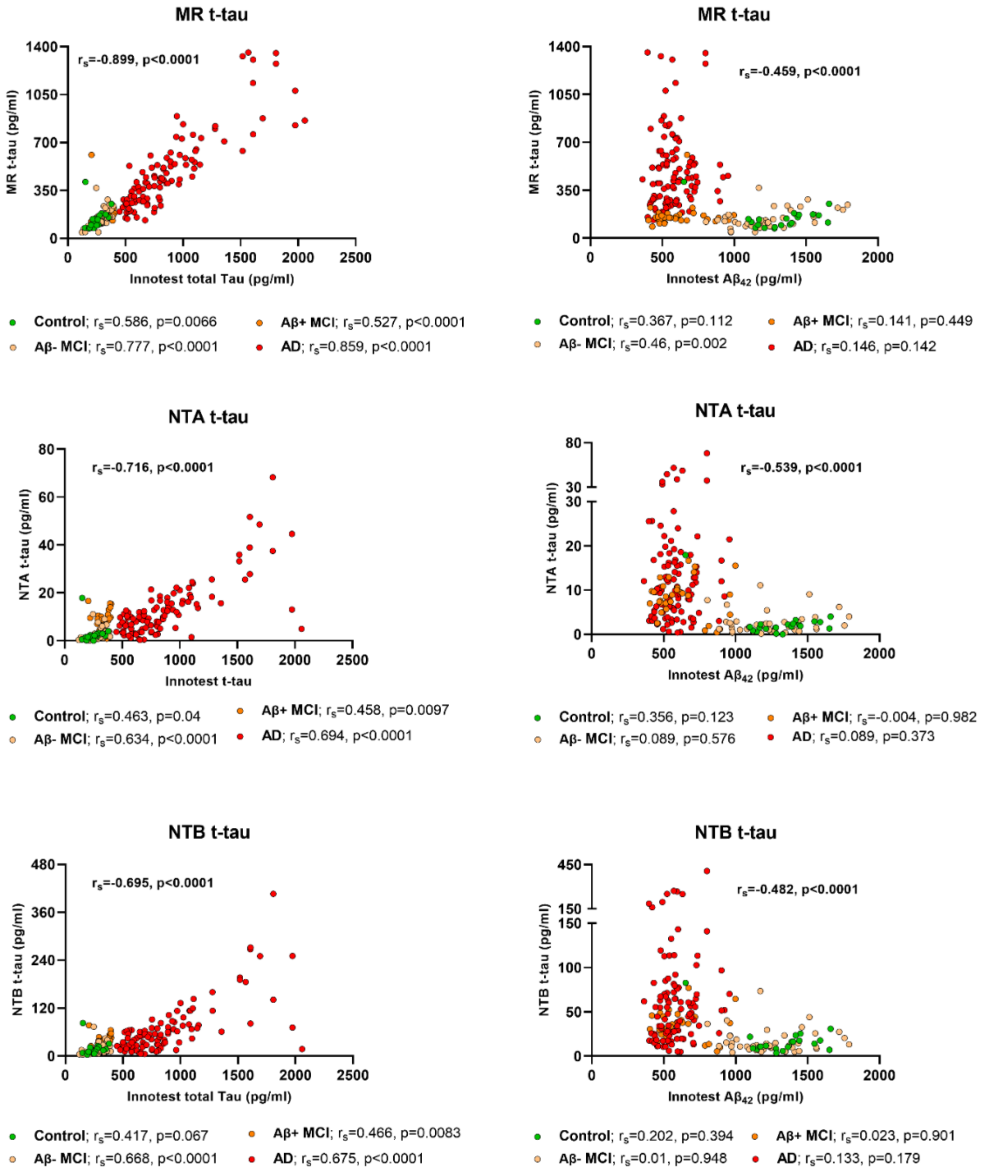

Supplementary Figure 3 Correlation of CSF MR, NTA, and NTB t-tau with Innotest CSF t-tau and CSF β-amyloid$_{1\text{-}42}$ (CSF clinical cohort 1)

Association between developed in-house CSF t-tau biomarkers with Innotest t-tau and Innotest amyloidβ$_{42}$ in the CSF clinical cohort 1 was evaluated using Spearman's correlation both in the whole cohort and within diagnostic groups.

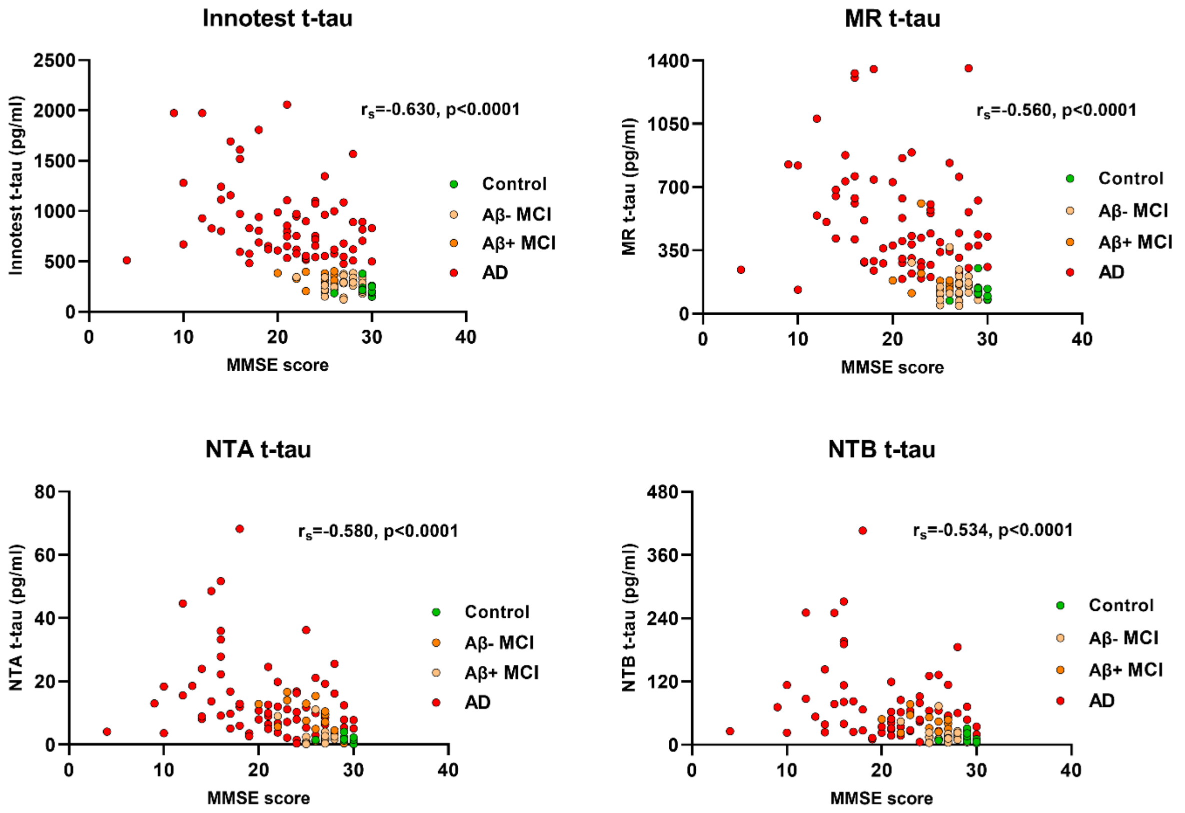

Supplementary Figure 4 Correlation of CSF t-tau biomarkers with Mini-Mental State Examination score (CSF clinical cohort 1)

Association between CSF t-tau biomarkers and Mini-mental state examination (MMSE) score was evaluated using Spearman's correlation in a sub-group of participants from the clinical CSF cohort 1. MMSE scores were available from 127/276 participants (Alzheimer's disease (AD), *n* = 72; amyloid-positive mild cognitive impairment (Aβ+ MCI), *n* = 16; amyloid negative (Aβ-) MCI, *n* = 28; controls, *n* = 11).

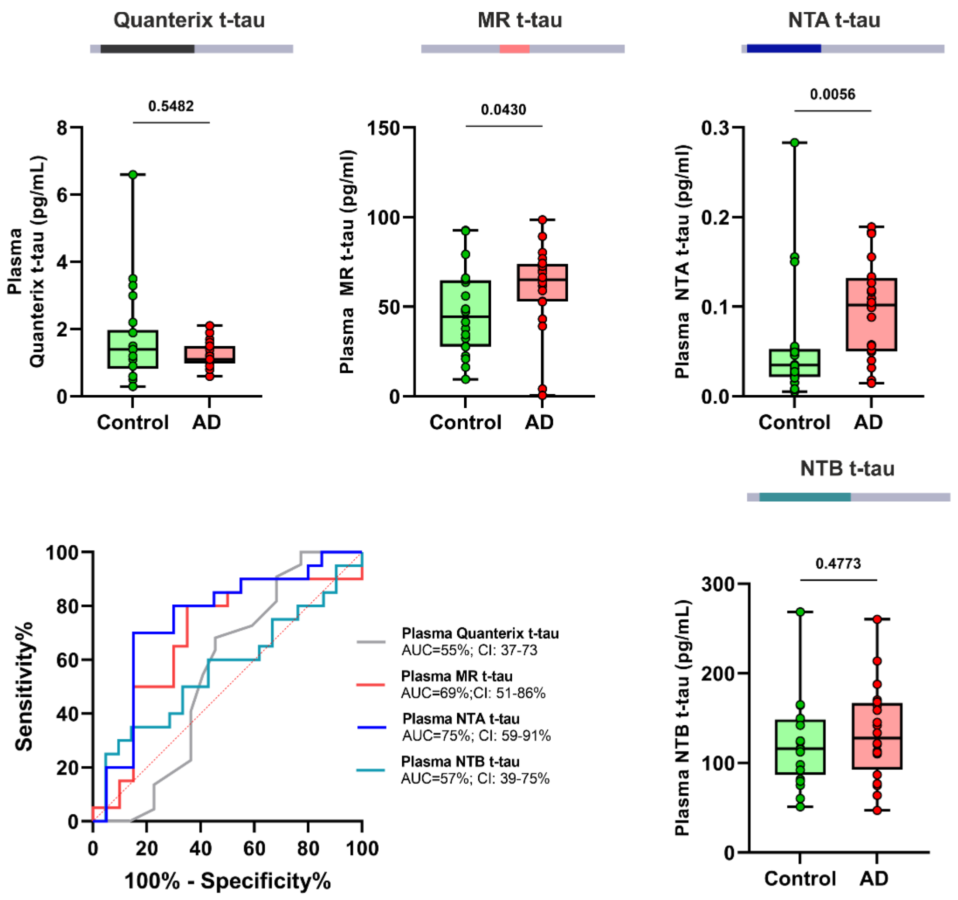

Supplementary Figure 5 Plasma concentrations and diagnostic performance of MR, NTA and NTB t-tau (Plasma pilot cohort)

Boxplots showing plasma MR t-tau, NTA t-tau and Quanterix t-tau concentrations in plasma pilot cohort including samples from biomarker-positive Alzheimer's disease patients (*n* = 20 for MR and NTA t-tau; *n* = 22 for NTB and Qanterix t-tau) and control patients (*n* = 20 for MR and NTA t-tau; *n* = 22 for NTB and Quanterix t-tau) assessed in the Sahlgrenska University Hospital, Gothenburg, Sweden. Due to limited sample availability, same set of samples was analysed with MR and NTA t-tau, and another set of samples with NTB and Quanterix t-tau (analyzed with single-analyte Tau 2.0 kit, #101552).

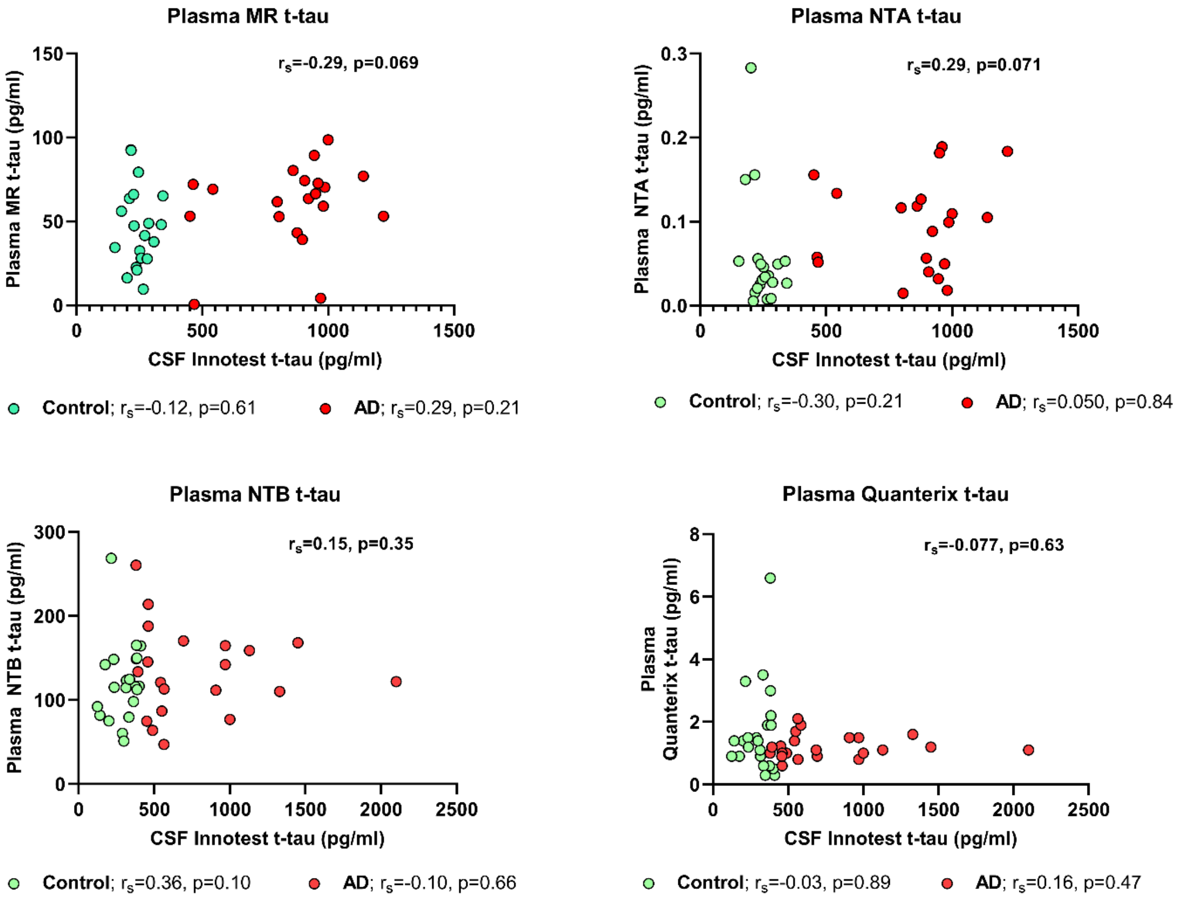

Supplementary Figure 6 Correlation of plasma MR, NTA, and NTB t-tau with CSF t-tau (Plasma pilot cohort).

Plasma pilot cohort included samples from biomarker-positive Alzheimer's disease patients (*n* = 20 for MR and NTA t-tau; *n* = 22 for NTB and Qanterix t-tau) and control patients (*n* = 20 for MR and NTA t-tau; *n* = 22 for NTB and Quanterix t-tau) assessed in the Sahlgrenska University Hospital, Gothenburg, Sweden. Groups were defined as Alzheimer's disease or control patients according to CSF biomarker profiles as described for the pilot CSF cohort. Association between plasma t-tau biomarkers and CSF t-tau (Innotest) was evaluated using Spearman's correlation.

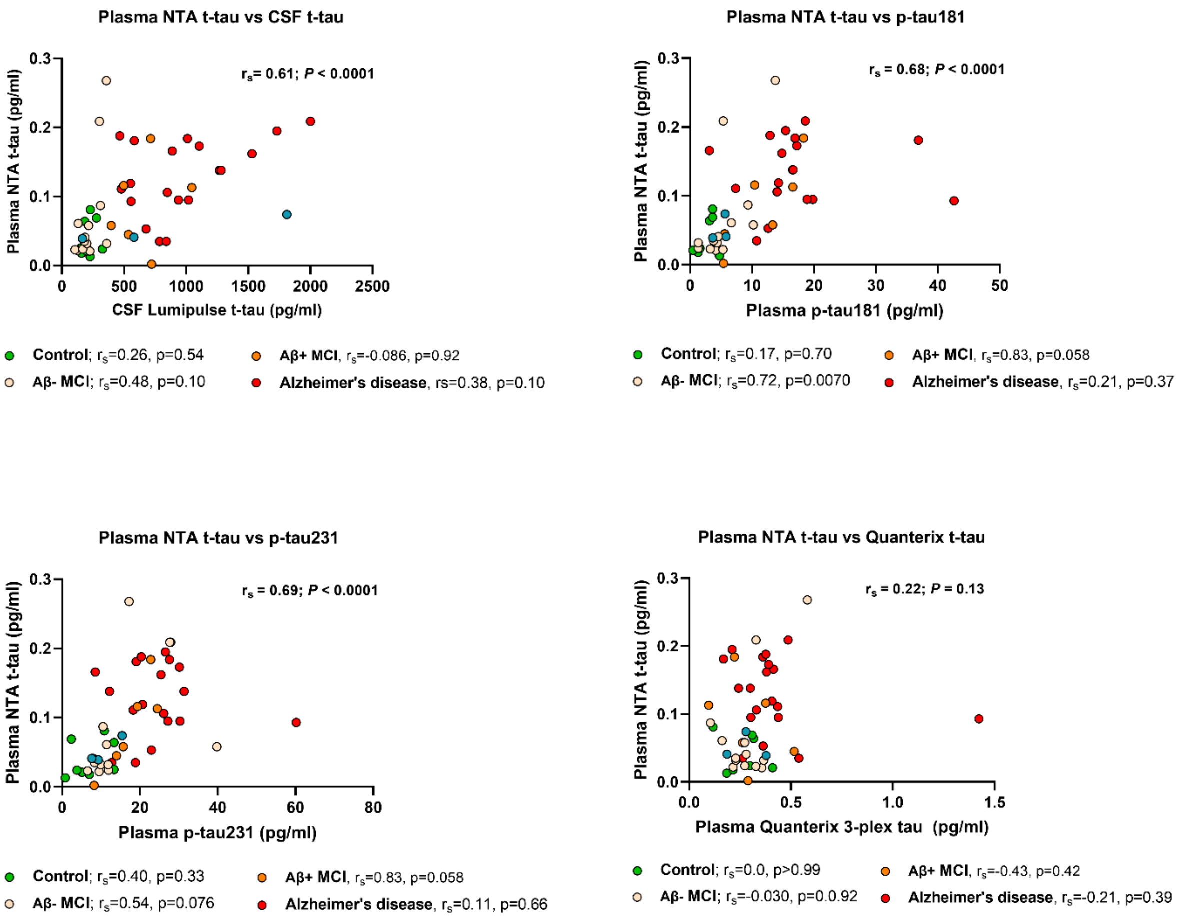

Supplementary Figure 7 Correlation of plasma NTA t-tau with CSF t-tau, plasma p-tau181, plasma p-tau231 and plasma Quanterix t-tau (Plasma clinical cohort).

The cohort included patients with Alzheimer's disease dementia (*n* = 19), amyloid positive (Aβ+) mild cognitive impairment (MCI) (*n* = 6), amyloid negative (Aβ-) MCI (*n* = 13), non-Alzheimer's disease dementia (*n* = 3) and controls (*n* = 8). Groups were defined according to their CSF biomarker profiles and neuropsychological assessment. Association between plasma t-tau biomarkers and CSF t-tau (Lumipulse) was evaluated using Spearman's correlation.
